# Deep neural networks excel in COVID-19 disease severity prediction—a meta-regression analysis

**DOI:** 10.1038/s41598-025-95282-6

**Published:** 2025-03-26

**Authors:** Márton Rakovics, Fanni Adél Meznerics, Péter Fehérvári, Tamás Kói, Dezső Csupor, András Bánvölgyi, Gabriella Anna Rapszky, Marie Anne Engh, Péter Hegyi, Andrea Harnos

**Affiliations:** 1 Centre for Translational Medicine, Semmelweis University, Budapest, Hungary; 2 Faculty of Social Sciences, Department of Statistics, ELTE Eötvös Loránd University, Budapest, Hungary; 3 Department of Dermatology, Venereology and Dermatooncology, Semmelweis University, Budapest, Hungary; 4 Biostatistics Department, University of Veterinary Medicine, Budapest, Hungary; 5 Department of Stochastics, Budapest University of Technology and Economics, Budapest, Hungary; 6 Institute of Clinical Pharmacy, University of Szeged, Szeged, Hungary; 7 Institute for Translational Medicine, Medical School, University of Pécs, Pécs, Hungary; 8 Institute of Pancreatic Diseases, Semmelweis University, Budapest, Hungary

**Keywords:** COVID-19, Severity prediction, Machine learning, Deep learning, Artificial intelligence, Viral infection, Prognosis, Prognostic markers, Risk factors, Statistics

## Abstract

COVID-19 is a disease in which early prognosis of severity is critical for desired patient outcomes and for the management of limited resources like intensive care unit beds and ventilation equipment. Many prognostic statistical tools have been developed for the prediction of disease severity, but it is still unclear which ones should be used in practice. We aim to guide clinicians in choosing the best available tools to make optimal decisions and assess their role in resource management and assess what can be learned from the COVID-19 scenario for development of prediction models in similar medical applications. Using the five major medical databases: MEDLINE (via PubMed), Embase, Cochrane Library (CENTRAL), Cochrane COVID-19 Study Register, and Scopus, we conducted a comprehensive systematic review of prediction tools between 2020 January and 2023 April for hospitalized COVID-19 patients. We identified both the relevant confounding factors of tool performance using the MetaForest algorithm and the best tools—comparing linear, machine learning, and deep learning methods—with mixed-effects meta-regression models. The risk of bias was evaluated using the PROBAST tool. Our systematic search identified eligible 27,312 studies, out of which 290 were eligible for data extraction, reporting on 430 independent evaluations of severity prediction tools with ~ 2.8 million patients. Neural Network-based tools have the highest performance with a pooled AUC of 0.893 (0.748–1.000), 0.752 (0.614–0.853) sensitivity, 0.914 (0.849–0.952) specificity, using clinical, laboratory, and imaging data. The relevant confounders of performance are the geographic region of patients, the rate of severe cases, and the use of C-Reactive Protein as input data. 88% of studies have a high risk of bias, mostly because of deficiencies in the data analysis. All investigated tools in use aid decision-making for COVID-19 severity prediction, but Machine Learning tools, specifically Neural Networks clearly outperform other methods, especially in cases when the basic characteristics of severe and non-severe patient groups are similar, and without the need for more data. When highly specific biomarkers are not available—such as in the case of COVID-19—practitioners should abandon general clinical severity scores and turn to disease specific Machine Learning tools.

**Supplementary Information:**

The online version contains supplementary material available at 10.1038/s41598-025-95282-6.

## Introduction

As of late 2024, over 700 M cases of coronavirus disease (COVID-19) have been registered, with around 7 M mortalities^1^ in connection with severe acute respiratory syndrome coronavirus-2 (SARS-CoV-2) infections. Respiratory symptoms are most commonly observed, but the disease may also affect the heart, kidneys, liver, pancreas, GI tract, brain, and blood vessels^2^. Infection-induced microangiopathy and microthrombi throughout the body also lead to multiorgan damage^3^.

Making a correct decision on the adequate level of care is critical for patients as the time from disease onset is a strong determinant of outcomes^4^ and is also crucial for the management of limited resources like intensive care unit (ICU) beds and ventilation equipment^5^. Many prognostic tools are in use for early prediction of disease severity, some of these being general clinical scores (e.g., MuLBSTA and CURB-65 ^6^. In contrast, others were developed specifically for COVID-19, ranging in methods from nomograms to artificial intelligence (AI) driven online applications^7^. The widespread use of vaccines and milder virus variants^8^ could not eliminate, only decrease the ratio of severe to total cases; thus, effective severity prediction remains essential.

There needs to be a consensus on which prognostic tools should be used in real-world situations. Our systematic review and meta-analysis quantifies the performance differences between currently available severity prediction tools to guide clinicians in making optimal decisions. We also aim to provide generalizable conclusions for developing severity prediction tools for other diseases, especially in resource-constrained scenarios.

As pulmonary complications are distinctive of COVID-19 and deep learning (DL) has been most successful in medical imaging^9^, we hypothesized that neural networks would outperform other methods in this application, especially the ones that incorporate imaging data.

Another important question related to this research is about the possible implications for model development in other diseases for similar severity prediction applications; thus, we investigate the relationship between the characteristics of patient groups and tool complexity to ascertain the requirements of different methods.

## Results

An overview of key results and corresponding methods are shown in Table 1.

**Table 1:** Summary of key results and methods.

Question	Method	Results
Which prediction tool has the highest performance?	Mixed-effects meta-regression and HSROC curves	Neural Networks have the highest AUC, and sensitivity for any given specificity. Machine learning methods have high performance even when the serve and non-severe patient groups have similar basic characteristics
What are the confounders for the relationship between tool type and performance?	MetaForest	Country of data collection, the rate of severe patients, and use of C-reactive protein as a biomarker are the most important determinants of performance along with tool type
What is the quality of synthetized studies?	PROBAST	Most studies have shortcomings in their data analysis and have limited generalizability

### Search and selection

**Fig. 1:**
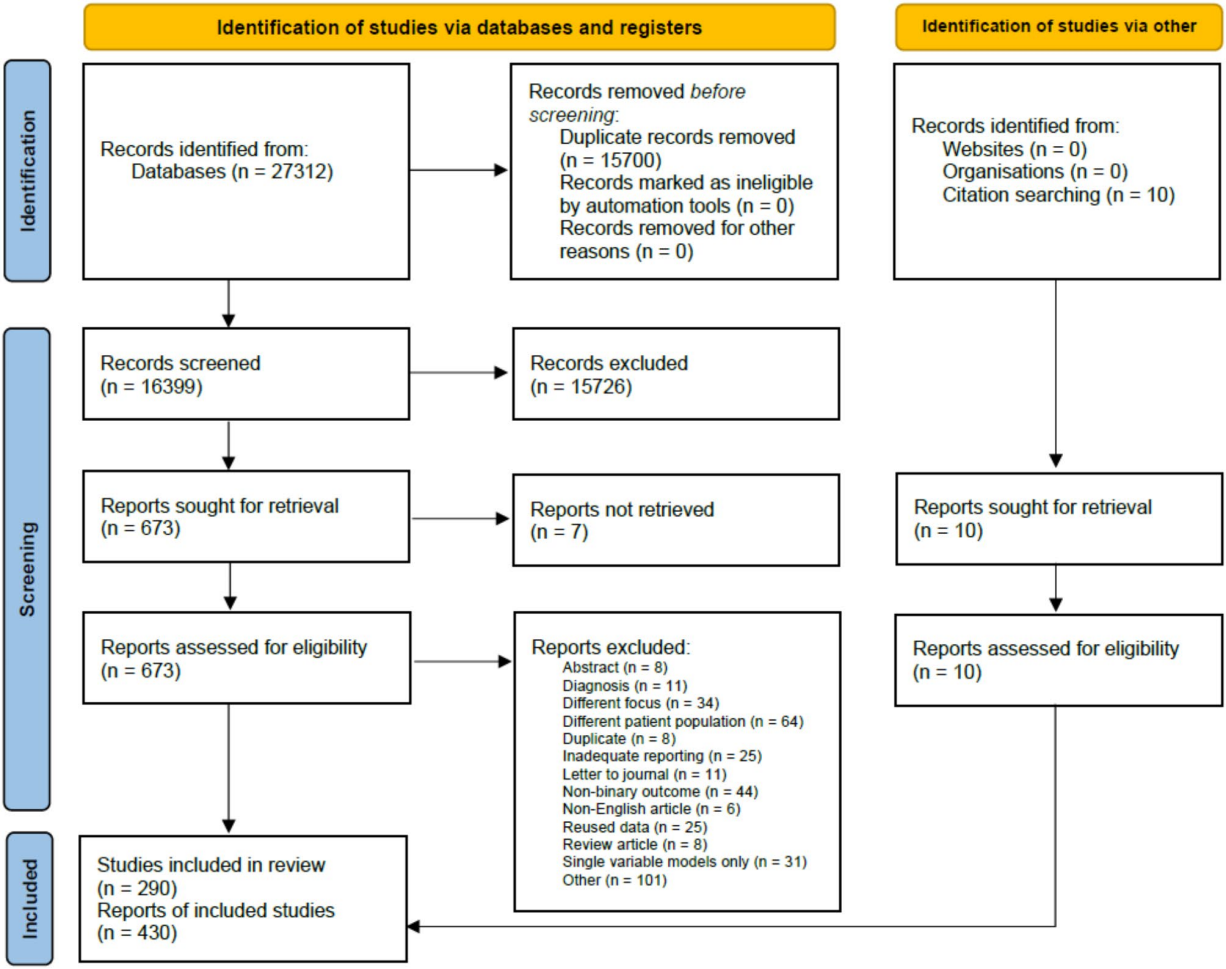
PRISMA 2020 flow diagram.

We identified 27,312 studies by systematically searching the five medical databases above (Medline: 13096, Embase: 5385, Cochrane and Cochrane Covid: 3411, Scopus: 5420). 16,399 records were screened, first based on the title and abstract, then on full texts. Ultimately, out of the 683 studies retrieved, 280 from database search and 10 from citations were eligible for data extraction. The selection process is summarised in Fig. 1. These 290 studies reported 430 independent evaluations of severity prediction tools.

### Descriptive characteristics of included studies

The 430 prediction tool evaluations used data from a total of 2,817,359 patients, with a mean sample size of 6,552 and a median of 320. Summary statistics for the three main tool types are given in Table 2; individual study characteristics are detailed in Supplementary Data S1, and references are listed in Supplementary References S1.

**Table 2:** Descriptive statistics for tool types.

Tool type	Number of studies	Ratio	AUC mean	AUC SD	AUC Q1	AUC Q3
Linear classifier	287	66.7%	0.851	0.081	0.799	0.911
Machine learning	102	23.7%	0.888	0.073	0.830	0.941
Neural network	41	9.5%	0.893	0.074	0.830	0.954

AUC: Area Under Receiver Operating Characteristic Curve

Of the 430 studies, 221 (51.4%) use mortality—either after 30 days of hospital admission or the final data collection date—to define severe cases and survivors as non-severe cases of COVID-19. Most of the other 209 studies use one of the following definitions: (1) moved to ICU, (2) needed some type of mechanical ventilation, or (3) the composite definition of the National Health Commission of the Peoples’s Republic of China^10^: having respiratory distress, respiratory rate ≥ 30 times/min, oxygen saturation ≤ 93%, PaO2/FiO2 ≤ 300 mm Hg. We grouped these non-mortality severe outcome definitions as none of the analyses showed substantial differences.

### Performance of severity prediction tools

From the 290 studies that provided enough data to assess Area Under receiver operating characteristic Curve (AUC), 430 independent evaluations could be extracted as many studies used different patient groups to develop and validate tools. 61.2% of tools are either preexisting clinical severity scores used as is or modified for COVID-19 or based on the simple logistic regression. Thus, most tools are linear classifiers that are easy to use and interpret, achieving a pooled AUC of 0.855 (SE 0.009) based on a random-effect meta-analysis. Without neural networks, machine learning tools have a pooled AUC of 0.891 (SE 0.008), while for neural network-based tools, the pooled AUC is 0.900 (SE 0.015). Tools in the latter two groups significantly (*p* < 0.001) outperform linear methods on average. However, substantial residual heterogeneity (I^2^ = 95.59%, and R^2^ = 5.84%) shows that other important factors affect performance besides the mathematical structure of the tools. (See detailed regression output in Supplementary Table S1 and results in Supplementary Table S2.)

### Identification of confounders of tool performance

In total, 24 potential variables were identified as possible confounders for measuring the effect of tool type on severity prediction performance. (The complete list of variables can be found in Supplementary Table S3.) Out of all candidates, 13 proved to have positive explained variance in out-of-bag samples in 99% of the 200 replications of the MetaForest model with 5000 trees: region of patient population, total number of patients included in the study, rate of severe patients, how severity was measured (as composite severity or as mortality) in the study, the time of the study, and the inclusion of age, C-reactive protein (CRP), respiratory rate, white blood count (WBC), measurement of blood gases, lactate dehydrogenase (LDH), blood urea nitrogen (BUN), and albumin as input variables of the prediction tool. Partial dependence plots suggested a quadratic relationship between AUC and the rate of severe patients in the study. (See replication importance values in Supplementary Fig. S1.)

### Area under the curve for severity prediction

After the preselection of confounders by the MetaForest algorithm, we used a mixed-effects meta-regression to estimate effect sizes and select a more parsimonious model by the permutation of the preselected variables. We opted for a model with 3 variables that retain an R^2^ of 35.12% compared to the full model’s 38.31%. As Table 3 shows, the 3 variables that have a significant effect are the region of the patient population, the rate of severe patients (and its squared value because of the quadratic dependence), and the inclusion of C-reactive protein (CRP) as an input variable of the prediction tool. (The results of the univariate analyses for the three confounders of AUC can be found in Supplementary Table S4.)

**Table 3:** Mixed-effects meta-regression results for AUC.

Variable	Category	Estimate	SE	t-value	d.f.	*p*-value
Tool type	Logistic regression - reference	0.857	0.010			
	Simple score	-0.029	0.012	-2.425	381	0.016
	Cox regression	-0.001	0.014	-0.033	381	0.979
	SVM	0.040	0.026	1.576	381	0.116
	Random forest	0.017	0.011	1.548	381	0.122
	Boosting	0.032	0.011	2.986	381	0.003
	Neural Network	0.039	0.020	1.956	381	0.051
	Deep Learning	0.024	0.014	1.680	381	0.094
Region	Europe (reference)					
	USA	-0.009	0.010	-0.886	381	0.376
	China	0.069	0.009	7.798	381	< 0.001
	Other region	0.006	0.009	0.660	381	0.510
Rate of severe cases		-0.146	0.027	-5.411	381	< 0.001
The rate of severe cases squared		0.478	0.103	4.649	381	< 0.001
CRP used	No (reference)					
	Yes	0.022	0.007	3.134	381	0.002

SVM: Support Vector Machine; Boosting: any tool that relies on boosting, e.g., XGBoost, or Gradient Boosting Machine; CRP: C-reactive protein; Rate of severe cases is centred at 25%.

As the reference setup, using a logistic regression prediction tool on European patients without CRP as input data and a 25% severity rate (the mean in collected data), the expected AUC is 0.857 (SE 0.010). A simple clinical score would have a significantly lower pooled AUC by 0.029 (SE 0.012), while neural network-based tools have a higher pooled AUC by 0.039 (SE 0.020). Thus, the difference in AUC between the best and worst tool types is 0.068 on average. For patients from China, prediction performance is better than that of Europeans by 0.069 (SE 0.009), and using CRP increases the expected pooled AUC by 0.022 (SE 0.007). To illustrate the magnitude of differences in performance, the expected AUC of a simple clinical score used in a European setting with 40% severity rate, not considering CRP, is 0.780 (SE 0.012), while a Neural Network in a Chinese setting with 5% severity rate and using CRP values is 0.998 (SE 0.020). The first tool is usually labelled fair, but the second is excellent^11^.

### Sensitivity and specificity of severity prediction

Studies are heterogeneous in their method of choosing sensitivity and specificity, but this is independent of tool type; thus, a comparison is valid. Values in Table 4 are adjusted for the region of the patient population, the rate of severe patients, and the inclusion of CRP as an input for the prediction. Because of insufficient reporting by the original studies, only 124 could be included in this analysis.

**Table 4:** Mean sensitivity and specificity by tool type, adjusted for confounders.

Tool type	Sensitivity (95% CI)	Specificity (95% CI)
Linear classifier	0.719 (0.632–0.792)	0.798 (0.726–0.855)
Machine Learning	0.781 (0.687–0.854)	0.831 (0.752–0.888)
Neural Network	0.752 (0.614–0.853)	0.914 (0.849–0.952)

**Fig. 2:**
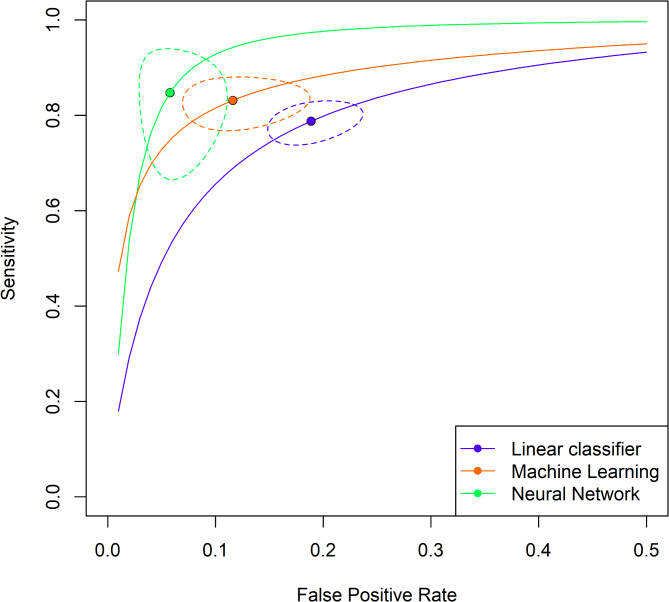
Study-level adjusted mean sensitivity and false positive rate. The figure shows the study-level adjusted mean sensitivity and false positive rate by tool type with 95% confidence interval for the mean and estimated hierarchical summary receiver operating characteristic curves (HSROC). (Note that the false positive rate goes up only to 50% on the figure.)

Supplementary Table S5 shows that the three tool types have significantly different mean sensitivity and specificity values. Linear classifiers are the least performant, with close to 72% sensitivity and 80% specificity. Meanwhile, neural network-based tools are the best, with a mean sensitivity and specificity of more than 75% and 91%, respectively. Study-level adjusted mean values are displayed in Fig. 2.

The same ordering of tool types can be observed: linear tools are the worst, machine learning tools are better, and neural networks are the best. Table 5 shows a hypothetical patient population of 700 million with 10% severe cases. Linear classifier-based tools give a positive test result for 271 M patients and correctly identify 44 M, machine learning tools produce 227 M positives, out of which 49 M are true positives. In contrast, Neural networks give a positive result for 144 M out of which 47 M are true positive cases.

**Table 5:** Estimated and adjusted performance metrics. The table shows the estimated and adjusted performance metrics for different tool types at a 10% patient severity rate in a patient population of size 700 M (equal to the total number of COVID-19 cases worldwide until late 2024).

Tool type	*N* true positive [M]	*N* false negative [M]	*N* true negative [M]	*N* false positive [M]	Sensitivity	Specificity	PPV	NPV
Linear classifier	44	26	403	227	0.632	0.640	0.163	0.940
Machine learning	49	21	453	177	0.706	0.718	0.218	0.957
Neural network	47	23	533	97	0.671	0.846	0.326	0.959

### Comparative analysis for tool development

#### Input modalities

Investigating the different types of input data modalities, we found that the ratio of tools that rely on only tabular data is 78.4%. Basic demographic data and laboratory measurements are used in about two-thirds of cases for both data types, 74.6% and 74.0%, respectively. Imaging data is used in 21.6% of cases, and none of the predictive tools incorporated text data. Using the mixed-effects meta-regression separately for the three main tool types, Table 6 shows that for linear methods, the use of laboratory data and imaging data both increase AUC, but only when one of these is used and not both, as indicated by the significance and effect size of the interaction term for lab and imaging. For machine learning or neural network-based tools, adding imaging or laboratory data to demographic and other clinical input data does not change the estimated pooled AUC significantly, suggesting that machine learning and neural network-based tools gain their classification performance benefit more from the non-linear mathematical structure than specific input data.

**Table 6:** Meta-regression estimates of the effects of input data types on AUC for the three tool types. The constant term is the estimated pooled AUC for a given tool type without imaging or laboratory data, and the interaction term is the additional effect of the two data types on AUC when both are used. Demographic and other clinical input data May be used in all cases.

Subset	Variable	Estimate	SE	*p*-value	Statistic	Value
Linear classifiers	No laboratory or imaging used	0.813	0.010	< 0.001	Tau2	0.005
	Imaging used	0.054	0.021	0.010	Tau2 SE	0.001
	Lab used	0.058	0.012	< 0.001	I^2^	94.66%
	Interaction of lab and imaging	-0.095	0.025	0.001	R^2^	9.93%
Machine learning	No laboratory or imaging used	0.870	0.020	< 0.001	Tau^2^	0.005
	Imaging used	-0.052	0.037	0.167	Tau^2^ SE	0.001
	Lab used	0.027	0.022	0.220	I^2^	95.83%
	Interaction of lab and imaging	0.067	0.043	0.119	R^2^	4.57%
Neural networks	No laboratory or imaging used	0.897	0.028	< 0.001	Tau^2^	0.005
	Imaging used	0.028	0.045	0.548	Tau^2^ SE	0.001
	Lab used	0.024	0.033	0.475	I^2^	95.64%
	Interaction of lab and imaging	-0.097	0.054	0.082	R^2^	8.49%

**Fig. 3:**
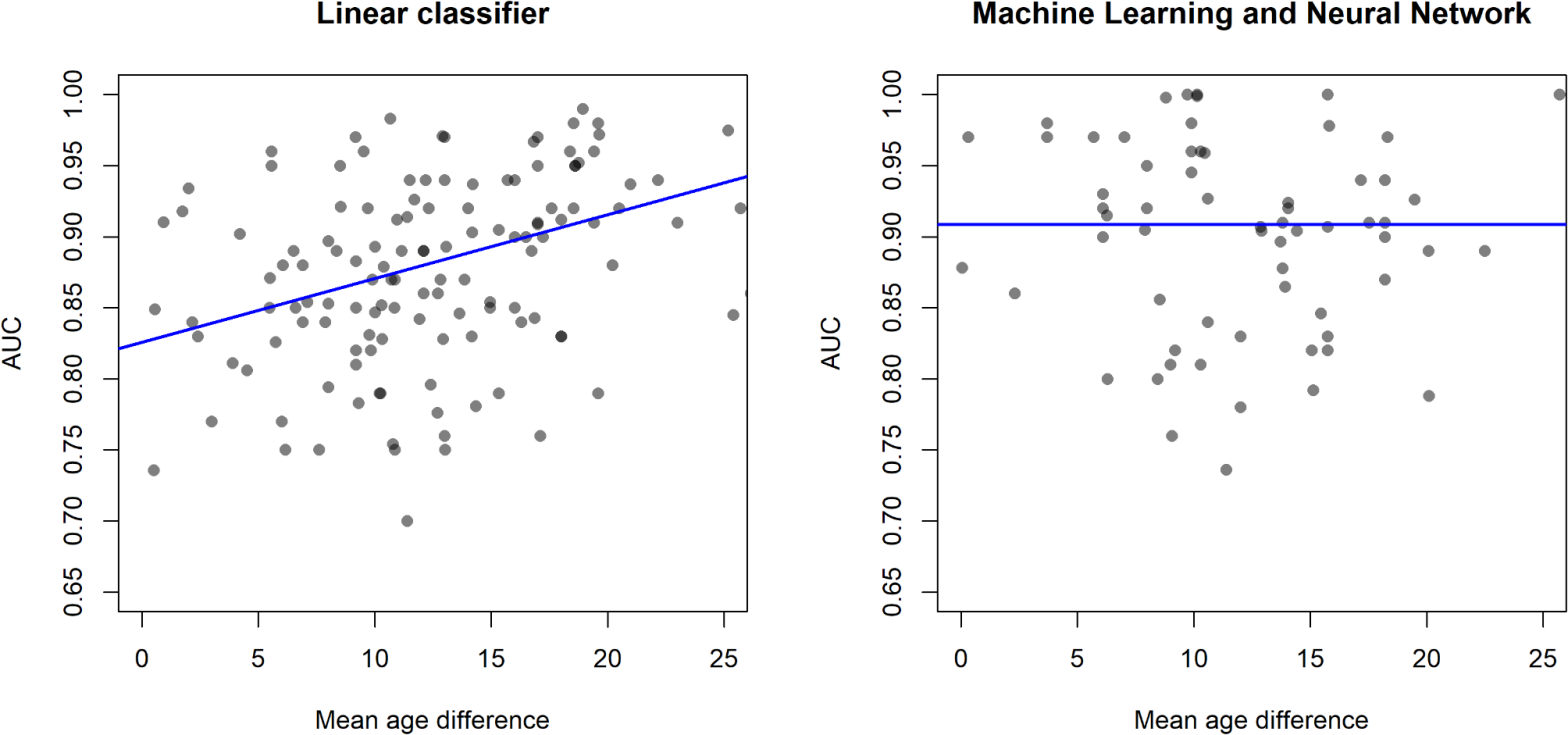
AUC by mean age difference for linear classifiers and machine learning/neural network tools separately. Blue lines show the estimated linear relationship, circles are studies.

We analysed AUC as a function of the mean age difference between severe and non-severe patient groups, where the latter serves as a proxy for the classification problem difficulty, including the number of patients, region, and rate of severe patients in the model as well. As mean age difference is an aggregate value, ecological bias may be present, but our separate patient-level analysis suggested this is not an issue. If the mean age difference is large, the two patient groups are easily separated by demographics, making the mathematical structure of the prediction tool or other input data less important. In such a setting, simple linear methods are expected to work well. As age is highly correlated with both disease severity and many clinical and laboratory measurements^12^, if the mean age difference is small, the two patient groups are not expected to be easily separable. Figure 3 shows that for linear tools, AUC is expected to significantly increase with an increase in mean age difference (slope of 0.0045 AUC/year), while machine learning or neural network-based tools do not display such a relationship. The number of patients in the dataset has a small but significant negative association with AUC for linear methods, which means prediction tools from larger studies have a lower performance. The dependence of AUC on the number of patients is not significant for machine learning and neural network-based tools, confirming that the increase in performance comes from the mathematical structure and not additional data.

131 studies had data for linear classifiers and 67 for machine learning tools. The estimated regression coefficients are in Table 7.

**Table 7:** Parameter estimates for mixed-effects meta-regression on AUC.

Subset	Variable	Value	Estimate	SE	*p*-value
Linear classifiers	Region	Europe - reference	0.826	0.029	< 0.001
		USA	-0.006	0.017	0.715
		China	0.057	0.013	< 0.001
		Other region	0.024	0.016	0.130
	Number of patients		-0.001	0.001	0.040
	Rate of severe cases		-0.196	0.168	0.247
	Rate of severe cases squared		0.241	0.289	0.406
	Mean age difference		0.005	0.001	< 0.001
Machine learning and Neural Network	Region	Europe - reference	0.908	0.029	< 0.001
		USA	0.020	0.023	0.381
		China	0.074	0.019	0.001
		Other region	-0.010	0.021	0.650
	Number of patients		-0.001	0.001	0.142
	Rate of severe cases		-0.189	0.162	0.250
	Rate of severe cases squared		0.168	0.231	0.470
	Mean age difference		< 0.001	0.001	0.785

#### Risk of bias assessment

**Fig. 4:**
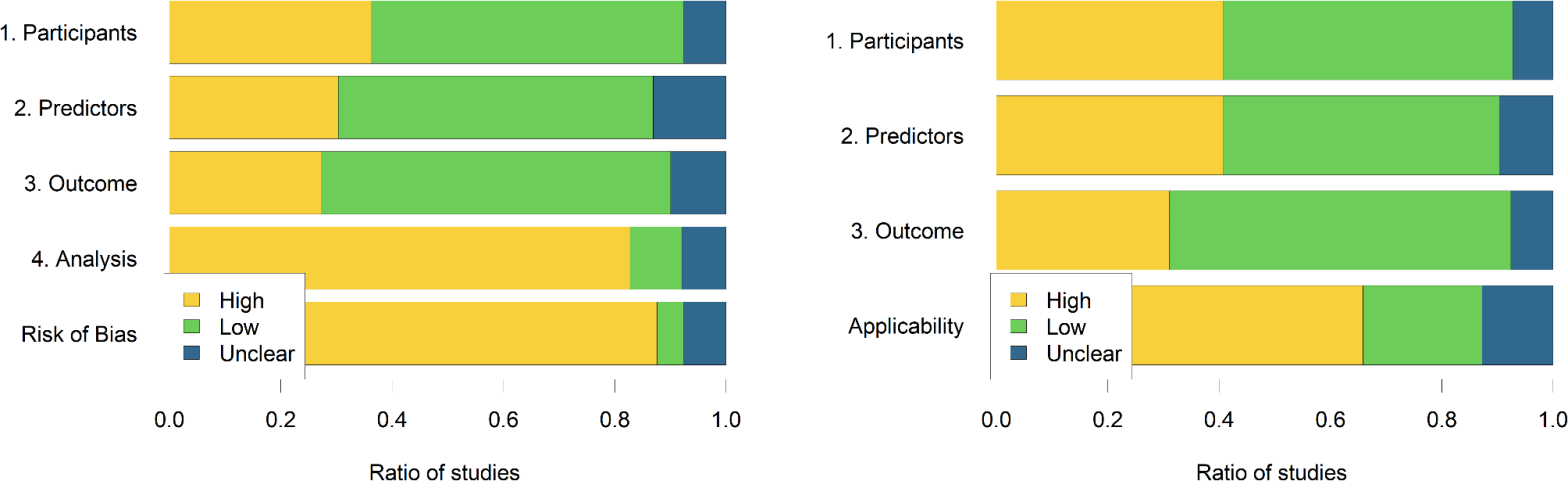
PROBAST results for Risk of Bias (left panel) and for Applicability (right panel).

The risk of bias and applicability assessment is shown in Fig. 4, as proposed by Moons et al.^13^. There is a high risk of bias overall for close to 88% of studies, predominantly because of the high risk of bias in the analysis phase. The two most often encountered errors in the analysis were (a) using univariate association measures between potential explanatory factors and the severity outcome to select variables to be included in a multivariate prediction tool and (b) *p*-value-driven variable selection. Handling missing data is also an issue, as most studies opt to exclude patients who have “too many” missing values, and even if missing values are imputed, detailed statistics comparing original and imputed values are not provided. Overfitting is primarily avoided by separating training and validation sets of patients, but performance metrics are only reported on separate test sets if data is gathered from multiple sources.

Supplementary Fig. S2 shows funnel plots for subgroups by main tool types and geographic region of the patient population, and corresponding Egger’s test results can be found in Supplementary Table S6.

## Discussion

Many prognostic tools have been developed for early prediction of disease severity in COVID-19, but which should be used in practice is an open question. We have conducted a systematic review and meta-analysis to quantify performance differences between severity prediction tools to aid clinicians in choosing the best options, and to assess what lessons can be learned for future prediction tool development for similar applications.

The MetaForest algorithm showed that after the geographic region of the study population, the second most important factor in predicting disease outcomes is the type of prediction tool itself. Machine learning tools outperform simple clinical scores and classical statistical models in all cases, even when sample size, study population characteristics, and other methodological choices are controlled for. Neural networks, in particular, have the highest pooled AUC of 0.893 (0.748–1.000), a sensitivity of 0.752 (0.614–0.853), and a specificity of 0.914 (0.849–0.952). Thus, clinicians’ default choice should not be the most often used simple scores and logistic regressions. The absence of clear sample size dependence of AUC suggests that if there is enough data for developing simple prediction tools, choosing an ML-based version is also possible, and has higher performance potential.

Using our original search key of “(Covid) AND (severity) AND (prediction OR prognosis)”, we identified 36 systematic reviews and meta-analyses on MEDLINE (via PubMed) up till 2023.07.01 the review of the association of demographic, clinical, laboratory measurements, medical imaging-based values, or comorbidities with disease severity. (For the list of review studies, see Supplementary References S2.) Out of these, five only consider comorbidities, while two cover only medical imaging. The top five measurements found to be associated with severity are D-Dimer, C-reactive protein (CRP), white blood cells (WBC), lactate dehydrogenase (LDH), and lymphocytes. Supplementary Table S7 shows the most important factors from the reviews with the ratio of prognostic tools using those factors. C-reactive protein is the only laboratory measurement that we considered in the final parsimonious meta-regression model, which was found to be the second most common factor associated with disease severity, showing to be a statistically significant prognostic feature in 51.7% of reviews.

We have also found four systematic reviews and meta-analyses focusing on severity prediction. Wynants et al.^14^ is an early review of available tools without a meta-analysis and highlights the fundamental problem of not following guidelines (e.g. TRIPOD, the transparent reporting of a multivariable prediction model for individual prognosis or diagnosis^15^) in reporting, which makes performance evaluations very difficult. de Jong et al.^16^ conducted an individual-level meta-analysis to validate previously developed prediction tools. They also found considerable heterogeneity in the performance of different tools but only considered simple logistic regression-based methods, omitting ML or DL tools altogether because of limited reproducibility. Wang et al.^17^ focus on deep learning tools for diagnosis and severity prediction using imaging data. Pooled sensitivity and specificity are 0.76 (95% CI 0.74–0.79) and 0.82 (95% CI 0.78–0.86) for prediction, which aligns with our findings. Chen et al.^18^ review 33 studies using machine learning tools (according to their definition of ML, which differs from the one used in our review). The pooled sensitivity for all studies is 0.86 (95% CI, 0.79–0.90), the specificity is 0.87 (95% CI, 0.80–0.92), and the AUC is 0.93 (95% CI, 0.90–0.95). These suggest better performance than our results, which can be attributed to their strict selection criteria (e.g., omitting studies with small sample sizes or without ML) that systematically favour tools with higher performance. Their list of the most important input variables for the prediction tools is highly similar to our list of variables found using the MetaForest selection algorithm, the top five being age, LDH, lymphocytes, respiratory rate, and CRP.

While our meta-analysis agrees with previous studies about the most important laboratory measurements, we could show that other more influential factors affect a prediction tool’s performance. The patient population plays a key role, at least through a regional effect and through the rate of severe cases. Prediction tools developed and used on European and North American patients were less precise than those on Chinese patients, and a lower rate of severe cases naturally meant that it was harder to accurately identify those patients. Additional analysis concerning the development of prediction tools suggested that using machine learning models is possible even without a large dataset and can handle cases with a complex relationship between measurements and severity categorization.

Our systematic review and meta-analysis is the most comprehensive to date. As we included an extensive range of COVID-19 disease severity prediction tools, the empirical foundation of our conclusions is more robust than that of previous reviews, which are all narrower in scope. Using the MetaForest algorithm, we could account for the relevant prognostic performance confounders, clearly showing ML tools’ superiority. While most previous reviews dealt with finding the best prediction factors, we could show that prognostic tools’ other features – notably the mathematical structure – have an even more critical role in determining performance.

A limitation for the generalizability of our results stems from the nature of COVID-19, as we have found that for such a systemic disease, there are many equally good sets of input measurements for prediction tools. Different tools based on information from clinical data, laboratory values, or medical imaging could all perform similarly well. For other diseases, finding a single most important biomarker out of many candidates or high reliance on medical imaging may prove the need for more specialized prediction tools.

A general implication for future research is that better adherence to reporting guidelines like TRIPOD^15^ is essential. Otherwise, it is tough to compare different methods and tools meticulously. Specifically, for COVID-19 severity prediction, as detailed imaging data does not have a predominant role here, the real strength of deep learning methods could not be utilized. However, when investigating the impact of patient demographics on performance, we found that simple linear methods worked well when the non-severe and the severe patient groups were clearly separable according to these, while machine learning tools performed well even if the two groups has similar basic characteristics.

It is well known that tree-based methods outperform neural networks for tabular data without specific changes to their architecture^19^. Both for new diseases and for new severity prediction tools in known ones, incorporating existing knowledge about the relationship between different measurements may be beneficial^9^. Graph-based deep learning methods^20^ could be viable options as the input graph structure can encode prior information about the features.

A potential drawback of using deep learning prediction tools is their limited interpretability compared to simple linear methods, but paired with auxiliary aids like adding SHAP values^21^ and interactive dashboards^22^ can mitigate this issue.

For practitioners, it is essential to understand that simple clinical scores may be useful if no distinct data is available for developing a disease-specific prognostic tool; substituting general prognostic tools for disease-specific ones should be done as quickly as possible because performance differences may be substantial. This highlights the importance of interdisciplinary cooperation, as put forth in the Cycle Model for Translational Medicine^23,24^, which argues that supporting a biostatistics team working alongside clinicians is essential in implementing state-of-the-art decision support tools.

All tools in use today aid decision-making for COVID-19 severity prediction, but machine learning tools, and specifically neural networks, clearly outperform other methods, most notably by decreasing the number of incorrectly identified severe cases and consequently helping a more efficient utilization of healthcare resources (e.g. ICU beds), which is critical in high-load scenarios like a pandemic. However, Deep learning radiomics tools are not required for this application, as image data seems redundant once clinical and laboratory measurements are available. When highly specific biomarkers are not available – such as in the case of COVID-19 – practitioners should abandon simple cutoff-based linear models (e.g. logistic regression) and general clinical severity scores (e.g. CURB-65 or NEWS2) as soon as possible and strive to develop task-specific machine learning tools.

## Methods

### Study design

This systematic review and meta-analysis is reported according to the PRISMA (Preferred Reporting Items for Systematic Reviews and Meta-Analyses) 2020 statement^25^ and follows Debray et al.’s A Guide to Systematic Review and Meta-analysis of Prediction Model Performance by the Cochrane Prognosis Methods Group^26,27^. We also considered the recommendations for the development, validation, and impact of statistical models that predict individual risk proposed by the PROGRESS (PROGnosis RESearch Strategy) partnership^28^ when evaluating the included studies, and the CHARMS checklist (CHecklist for critical Appraisal and data extraction for systematic Reviews of prediction Modelling Studies) which guides the reporting of prediction models^29^. The review protocol was registered on PROSPERO (registration number CRD42022377599, see www.crd.york.ac.uk/prospero).

### Information sources and eligibility criteria

The systematic literature search was conducted on 2023.04.02. in five major medical databases: MEDLINE (via PubMed), Embase, Cochrane Library (CENTRAL), Cochrane COVID-19 Study Register, and Scopus. Studies starting from 2020.01.01. up till the search date that reported on hospitalized adult patients with Reverse Transcription Polymerase Chain Reaction (RT-PCR) confirmed Sars-CoV-2 infection were included. The basis of comparison was using different severity prediction tools. In contrast, one of the outcomes measured needed to be dichotomous disease severity and to assess prediction tool performance, at least Area Under receiver operating characteristic Curve (AUC), sensitivity and specificity, or confusion matrix was required.

Studies reporting on paediatric, geriatric, or other sub-populations (e.g., patients with certain chronic diseases) were excluded, as well as those that only measured severity as a non-dichotomous quantity or exclusively investigated the role of a single factor of disease severity (e.g., the association of obesity with severity). Non-English articles, case reports/series, reviews, commentary articles, and letters to the editor were not considered. Only the original study was included whenever multiple studies analysed the same data.

### Search strategy

In all databases listed above, we used the simple search key of “(Covid) AND (severity) AND (prediction OR prognosis)” as the components of this are Medical Subject Headings (MeSH terms), which include relevant variations.

### Selection process

The selection was performed by two independent reviewers from the team (MR and GAR; Cohen’s Kappa = 0.92) for all studies after the duplicates were removed, first by title and abstract, then by assessing the full text. Disagreements were resolved by a third author (PF).

### Data collection process and data items

Based on a pilot data collection and the consensus of methodological and clinical experts, we created a standardized data collection sheet in Microsoft Excel. Data from all eligible articles were extracted independently by two authors. The data items collected were study period and geographical Area, study design, outcome definitions, patient demographics (group sizes, age, sex, country of origin), prediction tool type, statistical methods of variable selection, variables used in the prediction model, performance metrics (primary: AUC, secondary: sensitivity, specificity, positive predictive value, negative predictive value, confusion matrix entries).

### Study risk of bias and quality assessment

The risk of bias in the results and the applicability of the prognostic model were assessed using the PROBAST tool (Prediction model Risk of Bias Assessment Tool)^30^ by MR. The four domains for risk of bias assessment are participants, predictors, outcomes, and analysis; for applicability, participants, predictors, and outcomes. Each domain was classified as having a high, low, or unclear value.

### Synthesis methods

As the primary performance metric for comparing severity prediction tools, the Area Under the Receiver Operating Characteristic Curve (AUC) was pooled for tool types based on mathematical-statistical structure using a random-effects model^31^. The list of 8 types, from simpler toward more sophisticated models, are as follows: (a) clinical scores, (b) logistic and Cox regressions, (c) support vector machines (SVM), (d) random forests, (e) boosting models, (f) simple neural networks (single hidden-layer perceptron), (g) deep learning models (with at least two hidden-layers in the network). We also grouped types into 3 categories for some analyses: a-b as linear classifiers, types c-e as machine learning methods, and separately considering neural networks (f and g).

As secondary performance metrics, we analysed sensitivity and specificity values using a single point on the Receiver Operating Characteristic (ROC) curve as given in the original articles.

There are many possible confounding factors influencing tool performance (geographic region of study participants, number and types of demographic, clinical, laboratory, or other measurements used for prediction, etc.), which required a method that could perform an exploratory search and selection of relevant variables, while also accounted for between-study heterogeneity. The MetaForest method was selected as it was specifically developed for such problems^32^. It is a modification of the random forest algorithm by Breiman^33^, retaining its advantages in being robust against overfitting and capacity to model non-linear relationships, including higher-order interactions between the explanatory factors and the outcome performance metric. We used 200 replications with 5000 trees and random-effects weights, choosing the variables with positive estimates of explained variance in out-of-bag samples in 99% of cases.

First, we used MetaForest for AUC to find the important subset of control variables. Then we included these variables in a multivariate mixed-effects meta-regression model^34^, ensuring that no influential non-linear or interaction effects were excluded. Model checking was done using a QQ-plot for the residuals.

Although AUC is an important measure to compare tools and the one most commonly reported, the pair of sensitivity and specificity values are more characteristic of practical use as these refer to the actual operating point on the ROC curve while not being influenced by the true rate of severe patients. The same control variables were used when pooling sensitivity and specificity as for AUC. The bivariate model of Reitsma et al.^35,36^ was fitted, as this approach considers the dependency between sensitivity and specificity. The advantage of the analysis of the sensitivity and specificity is that these do not depend on the disease’s prevalence.

Positive predictive value (PPV) and negative predictive value (NPV) are more easily interpretable but depend on the rate of severe patients in the population. Nevertheless, we estimated positive predictive value (PPV) and negative predictive value (NPV) as a function of severity rate for different tool types. We hypothetically compared different tools in different application scenarios to show their influence on resource management.

The statistical analysis of the data was conducted using the R software (R Core Team, 2024, Vienna, Austria).

## Electronic supplementary material

Below is the link to the electronic supplementary material.

Supplementary Material 1

Supplementary Material 2

Supplementary Material 3

## Data Availability

All data used in this study can be found in the full-text articles included in the systematic review and meta-analysis. Data is provided within the supplementary information files.
