# Supplementary material for: Deep neural networks excel in COVID-19 disease severity prediction—a meta-regression analysis
Source: Sci Rep. 2025 Mar 26;15:10350. doi: 10.1038/s41598-025-95282-6 (PMC11937321; doi:10.1038/s41598-025-95282-6)
Supplement: Supplementary file 3 — Supplementary Material 3 [file 41598_2025_95282_MOESM3_ESM.pdf]

Deep Neural Networks Excel in COVID-19 Disease Severity Prediction – a Meta-Regression Analysis

Márton Rakovics[1,2,*], Fanni Adél Meznerics[1,3], Péter Fehérvári[1,4], Tamás Kói[1,5], Dezső Csupor[1,6,7], András Bánvölgyi[1,3], Gabriella Anna Rapszky[1], Marie Anne Engh[1], Péter Hegyi[1,7,8], Andrea Harnos[1,4]

[1] Centre for Translational Medicine, Semmelweis University, Budapest, Hungary.
[2] ELTE Eötvös Loránd University, Faculty of Social Sciences, Department of Statistics, Budapest, Hungary.
[3] Department of Dermatology, Venereology and Dermatooncology, Semmelweis University, Budapest, Hungary.
[4] University of Veterinary Medicine, Biostatistics Department, Budapest, Hungary.
[5] Budapest University of Technology and Economics, Department of Stochastics, Budapest, Hungary.
[6] Institute of Clinical Pharmacy, University of Szeged, Szeged, Hungary.
[7] Institute for Translational Medicine, Medical School, University of Pécs, Pécs, Hungary.
[8] Institute of Pancreatic Diseases, Semmelweis University, Budapest, Hungary.

* Corresponding author: Márton Rakovics (marton.rakovics@tatk.elte.hu).

Supplementary Information

Supplementary Tables

Supplementary Table S1. Random-effects meta-analysis for tool types.

Category	Estimate	SE	t-value	d.f.	p-value	CI lower	CI upper
Machine Learning – reference category	0.8909	0.0078	113.5322	401	< 0.001	0.8755	0.9063
Linear classifier	-0.0362	0.0091	-3.9558	401	< 0.001	-0.0541	-0.0182
Neural Network	0.0089	0.0148	0.6014	401	0.5479	-0.0202	0.038

Supplementary Table S2. Relevant statistics to Table 1.

Statistic	Value	SE
Tau^2 (estimated amount of residual heterogeneity)	0.0053	0.0004
I^2 (residual heterogeneity)	95.59%	
R^2 (amount of heterogeneity accounted for)	5.84%	

Supplementary Table S3. Complete list of variables and replication importance values.

List of variables	Values and description
tool type	Categorical variable with 8 categories: (a) clinical scores, (b) logistic and Cox regressions, (c) support vector machines, (d) random forests, (e) boosting models, (f) simple neural networks, (g) deep learning models.
region	Categorical variable: Europe, USA, China, other region.
timeframe	Year of most recent case in the dataset.

outcome type	Binary: Non-severe (0), or Severe (1). The definition of severity is detailed in the main text.
severity rate	Rate of severe patients in the study.
deep learning (DL) imaging features	Binary: Imaging information from a deep learning model is directly included (1) or not in the prediction tool (0).
sample size	Number of patients in the dataset used for the given prognostic tool (training, validation, or test set considered separately).
imaging	Binary: Any type of imaging information included (1) or not included (0) in the prediction tool.
comorbidities	Binary: Any type of information on comorbidities included (1) or not included (0) in the prediction tool.
age	Binary: Age of patient included (1) or not included (0) in the prediction tool.
sex	Binary: Sex of patient included (1) or not included (0) in the prediction tool.
C-reactive protein (CRP)	Binary: Measurement included (1) or not included (0) in the prediction tool.
lymphocytes	Binary: Measurement included (1) or not included (0) in the prediction tool.
D-dimer	Binary: Measurement included (1) or not included (0) in the prediction tool.
neutrophils	Binary: Measurement included (1) or not included (0) in the prediction tool.
CT	Binary: Measurement included (1) or not included (0) in the prediction tool.
lactate dehydrogenase (LDH)	Binary: Measurement included (1) or not included (0) in the prediction tool.
blood gases	Binary: Measurement included (1) or not included (0) in the prediction tool.
respiratory rate	Binary: Measurement included (1) or not included (0) in the prediction tool.
blood urea nitrogen (BUN)	Binary: Measurement included (1) or not included (0) in the prediction tool.
albumin	Binary: Measurement included (1) or not included (0) in the prediction tool.
platelets	Binary: Measurement included (1) or not included (0) in the prediction tool.
white blood cells (WBC)	Binary: Measurement included (1) or not included (0) in the prediction tool.
creatinine	Binary: Measurement included (1) or not included (0) in the prediction tool.
blood pressure	Binary: Measurement included (1) or not included (0) in the prediction tool.

Supplementary Table S4. Univariate random-effects meta-analysis for AUC for the three important confounders in the multivariate regression model.

Variable	Category	Estimate	SE	t-value	d.f.	p-value
Region	Europe - reference	0.837	0.007	128.649	400	<0.001
	USA	0.004	0.011	0.400	400	0.689
	China	0.082	0.009	9.020	400	<0.001
	Other region	0.020	0.010	2.058	400	0.04
Severity rate	Constant	0.900	0.007	122.359	392	<0.001
	Severity rate	-0.179	0.030	-6.061	392	<0.001
	Severity rate squared	0.559	0.114	4.889	392	<0.001
CRP	CRP not used - reference	0.857	0.005	164.862	402	<0.001
	CRP used	0.023	0.008	2.955	402	0.003

Supplementary Table S5. Pairwise comparisons of sensitivity and specificity between tool types using Hotelling's T2 tests.

Comparison	T^2-value	d.f.	p-value
Linear classifier vs. Machine Learning	153.34	2; 70.44	< 0.001
Linear classifier vs. Neural Network	454.61	2; 24.26	< 0.001
Machine Learning vs. Neural Network	172.47	2; 31.47	< 0.001

Supplementary Table S6. Corresponding Egger's test results to the funnels plots. Note that Egger's test results may not be reliable in this case, since AUC and its standard error is correlated with sample size.

Tool	Region	t-value	d.f.	p-value
Linear classifier	Europe	-1.796	82	0.076
Machine Learning	Europe	-0.414	26	0.682
Neural Network	Europe	-3.795	5	0.012
Linear classifier	USA	-0.845	38	0.402
Machine Learning	USA	-2.263	16	0.030
Neural Network	USA	0.220	5	0.833
Linear classifier	China	-3.309	84	0.001
Machine Learning	China	-2.023	24	0.050
Neural Network	China	-1.823	14	0.089
Linear classifier	Other region	-1.353	60	0.181
Machine Learning	Other region	-2.641	21	0.015
Neural Network	Other region	-0.759	5	0.481

Supplementary Table S7. Top five variables from previous systematic reviews.

Variable	Ratio of reviews showing significant association	Ratio in prognostic tools using the variable
D-Dimer	69.0%	15.1%
CRP	51.7%	42.3%
WBC	48.3%	18.6%
LDH	41.4%	27.0%
Lymphocytes	37.9%	32.3%

CRP: C-reactive protein, WBC: white blood cells, LDH: lactate dehydrogenase.

Supplementary Figures

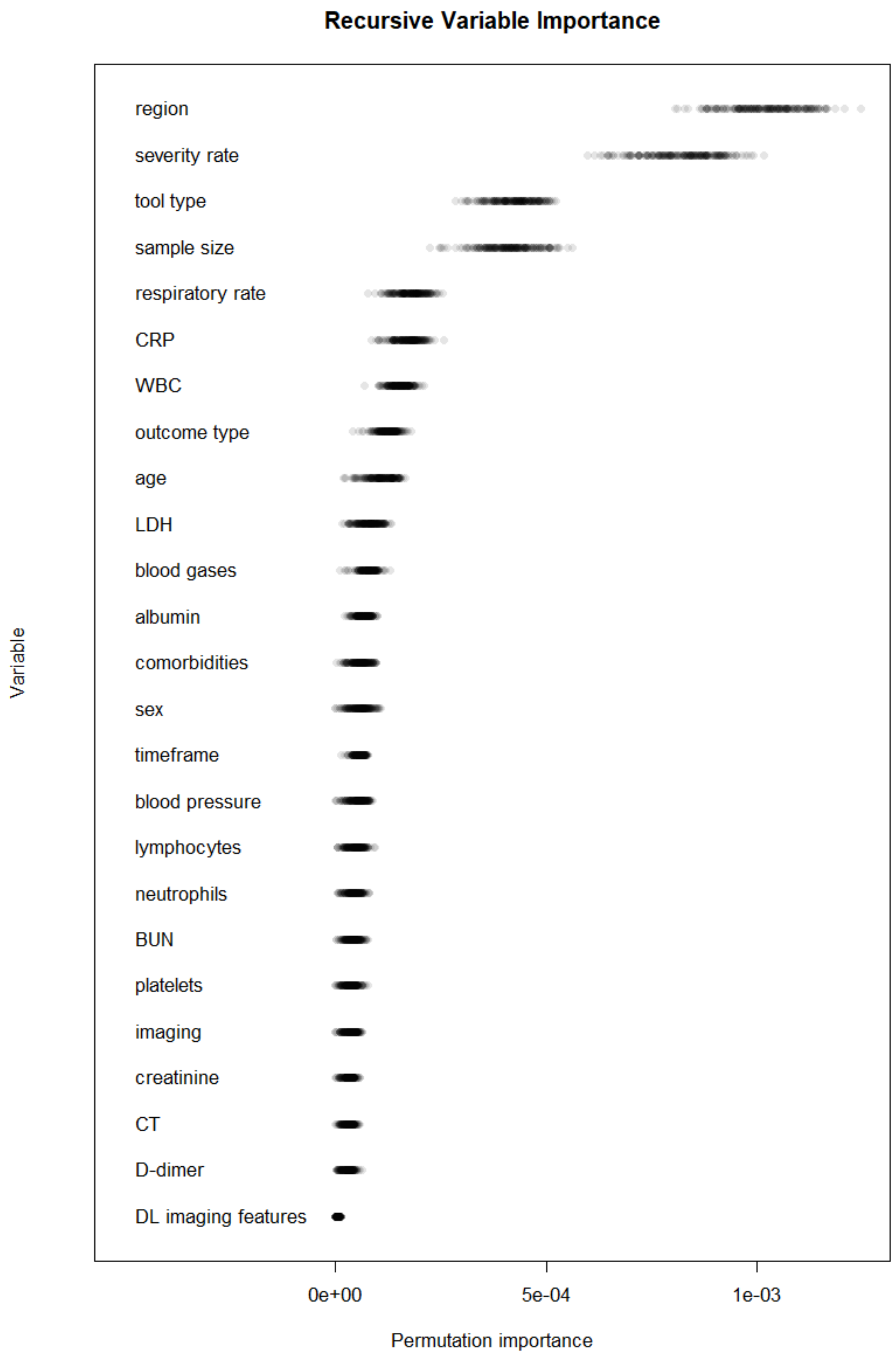

Supplementary Figure S1. Permutation variable importance values.

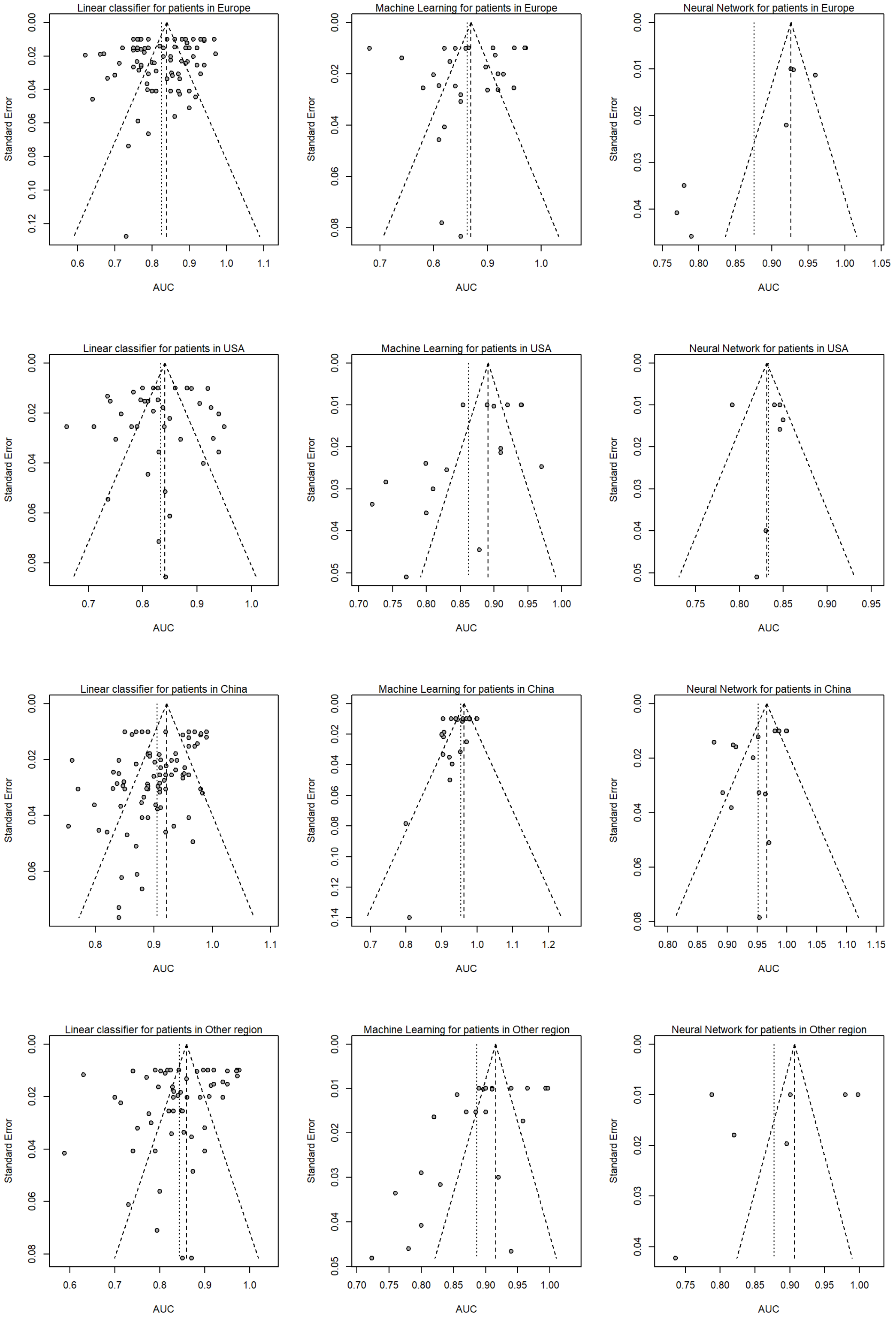

Supplementary Figure S2. Funnel plots for subgroups by main tool types and geographic region of patient population.

Supplementary References

Supplementary Reference S1. List of studies included in the meta-analysis.

1.
Jamal, M. H. *et al.* A biomarker based severity progression indicator for COVID-19: the Kuwait prognosis indicator score. *Biomarkers* **25**, 641–648 (2020).
2.
Israel, A. *et al.* A Calculator for COVID-19 Severity Prediction Based on Patient Risk Factors and Number of Vaccines Received. *Microorganisms* **10**, 1238 (2022).
3.
Galloway, J. B. *et al.* A clinical risk score to identify patients with COVID-19 at high risk of critical care admission or death: An observational cohort study. *J Infect* **81**, 282–288 (2020).
4.
Hong, W. *et al.* A Comparison of XGBoost, Random Forest, and Nomograph for the Prediction of Disease Severity in Patients With COVID-19 Pneumonia: Implications of Cytokine and Immune Cell Profile. *Front Cell Infect Microbiol* **12**, 819267 (2022).
5.
Xu, J. *et al.* A composite risk model predicts disease progression in early stages of COVID-19: A propensity score-matched cohort study. *Ann Clin Biochem* **58**, 434–444 (2021).
6.
Aloisio, E. *et al.* A Comprehensive Appraisal of Laboratory Biochemistry Tests as Major Predictors of COVID-19 Severity. *Archives of Pathology & Laboratory Medicine* **144**, 1457–1464 (2020).
7.
Meng, L. *et al.* A Deep Learning Prognosis Model Help Alert for COVID-19 Patients at High-Risk of Death: A Multi-Center Study. *IEEE J Biomed Health Inform* **24**, 3576–3584 (2020).
8.
Shi, W. *et al.* A deep learning-based quantitative computed tomography model for predicting the severity of COVID-19: a retrospective study of 196 patients. *Ann Transl Med* **9**, 216 (2021).
9.
Chieregato, M. *et al.* A hybrid machine learning/deep learning COVID-19 severity predictive model from CT images and clinical data. *Sci Rep* **12**, 4329 (2022).
10.
Mahdavi, M. *et al.* A machine learning based exploration of COVID-19 mortality risk. *PLoS One* **16**, e0252384 (2021).
11.
Bolourani, S. *et al.* A Machine Learning Prediction Model of Respiratory Failure Within 48 Hours of Patient Admission for COVID-19: Model Development and Validation. *Journal of Medical Internet Research* **23**, e24246 (2021).
12.
Gong, K. *et al.* A multi-center study of COVID-19 patient prognosis using deep learning-based CT image analysis and electronic health records. *Eur J Radiol* **139**, 109583 (2021).
13.
Pezoulas, V. C. *et al.* A Multimodal Approach for the Risk Prediction of Intensive Care and Mortality in Patients with COVID-19. *Diagnostics (Basel)* **12**, 56 (2021).

14.
Wang, Z. *et al.* A multiplex protein panel assay for severity prediction and outcome prognosis in patients with COVID-19: An observational multi-cohort study. *EClinicalMedicine* **49**, 101495 (2022).
15.
Sharifi, M., Khademian, M. H., Mousavi-Roknabadi, R. S., Ebrahimi, V. & Sadegh, R. A New Rapid Approach for Predicting Death in Coronavirus Patients: The Development and Validation of the COVID-19 Risk-Score in Fars Province (CRSF). *Iran J Public Health* **51**, 178–187 (2022).
16.
Liu, S. *et al.* A nomogram predicting severe COVID-19 based on a large study cohort from China. *Am J Emerg Med* **50**, 218–223 (2021).
17.
Çelikkol, A., Doğan, M., Güzel, E. C., Erdal, B. & Yılmaz, A. A Novel Combined Index of D-Dimer, Fibrinogen, Albumin, and Platelet (FDAPR) as Mortality Predictor of COVID-19. *Nigerian Journal of Clinical Practice* **25**, 1418 (2022).
18.
Mishra, P. *et al.* A novel epidemiological scoring system for the prediction of mortality in COVID-19 patients. *Trans R Soc Trop Med Hyg* **116**, 409–416 (2022).
19.
Linssen, J. *et al.* A novel haemocytometric COVID-19 prognostic score developed and validated in an observational multicentre European hospital-based study. *Elife* **9**, e63195 (2020).
20.
Xu, W. *et al.* A Novel Prediction Model of COVID-19 Progression: A Retrospective Cohort Study. *Infect Dis Ther* **10**, 1491–1504 (2021).
21.
Detsika, M. G. *et al.* A novel ratio of CD8+:B-cells as a prognostic marker of coronavirus disease 2019 patient progression and outcome. *Virology* **556**, 79–86 (2021).
22.
Amirzadehfard, F. *et al.* A Novel Scoring System for Early Assessment of the Risk of the COVID-19-associated Mortality in Hospitalized Patients: COVID-19 BURDEN. 2022.01.09.22268975 Preprint at https://doi.org/10.1101/2022.01.09.22268975 (2022).
23.
Zhang, C. *et al.* A Novel Scoring System for Prediction of Disease Severity in COVID-19. *Front Cell Infect Microbiol* **10**, 318 (2020).
24.
Altschul, D. J. *et al.* A novel severity score to predict inpatient mortality in COVID-19 patients. *Sci Rep* **10**, 16726 (2020).
25.
Dong, Y. *et al.* A novel simple scoring model for predicting severity of patients with SARS-CoV-2 infection. *Transbound Emerg Dis* **67**, 2823–2829 (2020).
26.
Sia, T. L. L. *et al.* A pre-admission triaging tool to predict severe COVID-19 cases: ABCD score. *Med J Malaysia* **77**, 237–240 (2022).
27.

Lalueza, A. *et al.* A predictive score at admission for respiratory failure among hospitalized patients with confirmed 2019 Coronavirus Disease: a simple tool for a complex problem. *Intern Emerg Med* **17**, 515–524 (2022).
28.
Xu, J. *et al.* A predictive score for progression of COVID-19 in hospitalized persons: a cohort study. *NPJ Prim Care Respir Med* **31**, 33 (2021).
29.
Nagant, C. *et al.* A score combining early detection of cytokines accurately predicts COVID-19 severity and intensive care unit transfer. *Int J Infect Dis* **101**, 342–345 (2020).
30.
Elhendawy, M. *et al.* A Simple Scoring Model Predicting the Outcome of COVID-19 Patients: Tanta COVID Score. *Endocr Metab Immune Disord Drug Targets* **22**, 620–630 (2022).
31.
Gong, J. *et al.* A Tool for Early Prediction of Severe Coronavirus Disease 2019 (COVID-19): A Multicenter Study Using the Risk Nomogram in Wuhan and Guangdong, China. *Clin Infect Dis* **71**, 833–840 (2020).
32.
Marcolino, M. S. *et al.* ABC2-SPH risk score for in-hospital mortality in COVID-19 patients: development, external validation and comparison with other available scores. *Int J Infect Dis* **110**, 281–308 (2021).
33.
Zou, X. *et al.* Acute Physiology and Chronic Health Evaluation II Score as a Predictor of Hospital Mortality in Patients of Coronavirus Disease 2019. *Crit Care Med* **48**, e657–e665 (2020).
34.
Soda, P. *et al.* AIforCOVID: Predicting the clinical outcomes in patients with COVID-19 applying AI to chest-X-rays. An Italian multicentre study. *Med Image Anal* **74**, 102216 (2021).
35.
Zhang, M. *et al.* An AI-based radiomics nomogram for disease prognosis in patients with COVID-19 pneumonia using initial CT images and clinical indicators. *Int J Med Inform* **154**, 104545 (2021).
36.
Chiari, M. *et al.* An Application of Recurrent Neural Networks for Estimating the Prognosis of COVID-19 Patients in Northern Italy. in *Artificial Intelligence in Medicine* (eds. Tucker, A., Henriques Abreu, P., Cardoso, J., Pereira Rodrigues, P. & Riaño, D.) 318–328 (Springer International Publishing, Cham, 2021). doi:10.1007/978-3-030-77211-6_36.
37.
Nanivadekar, A. *et al.* An artificial intelligence system for predicting mortality in COVID-19 patients using chest X-rays: a retrospective study. 2021.09.22.21263956 Preprint at https://doi.org/10.1101/2021.09.22.21263956 (2021).
38.
Chowdhury, M. E. H. *et al.* An Early Warning Tool for Predicting Mortality Risk of COVID-19 Patients Using Machine Learning. *Cogn Comput* (2021) doi:10.1007/s12559-020-09812-7.
39.
Kim, H.-J. *et al.* An Easy-to-Use Machine Learning Model to Predict the Prognosis of Patients With COVID-19: Retrospective Cohort Study. *J Med Internet Res* **22**, e24225 (2020).

40.
Acar, H. C. *et al.* An easy-to-use nomogram for predicting in-hospital mortality risk in COVID-19: a retrospective cohort study in a university hospital. *BMC Infectious Diseases* **21**, 148 (2021).
41.
Zheng, B. *et al.* An Interpretable Model-Based Prediction of Severity and Crucial Factors in Patients with COVID-19. *Biomed Res Int* **2021**, 8840835 (2021).
42.
Sinha, A., Joshi, S. P., Das, P. S., Jana, S. & Sarkar, R. An ML prediction model based on clinical parameters and automated CT scan features for COVID-19 patients. *Sci Rep* **12**, 11255 (2022).
43.
Weng, Z. *et al.* ANDC: an early warning score to predict mortality risk for patients with Coronavirus Disease 2019. *J Transl Med* **18**, 328 (2020).
44.
Ma, B. *et al.* Applicability of MuLBSTA scoring system as diagnostic and prognostic role in early warning of severe COVID-19. *Microb Pathog* **150**, 104706 (2021).
45.
Ye, J., Zhang, X., Zhu, F. & Tang, Y. Application of a prediction model with laboratory indexes in the risk stratification of patients with COVID-19. *Exp Ther Med* **21**, 182 (2021).
46.
Erturk Sengel, B. *et al.* Application of CALL score for prediction of progression risk in patients with COVID-19 at university hospital in Turkey. *Int J Clin Pract* **75**, e14642 (2021).
47.
Wang, R. *et al.* Artificial intelligence for prediction of COVID-19 progression using CT imaging and clinical data. *Eur Radiol* **32**, 205–212 (2022).
48.
Esposito, A. *et al.* Artificial Intelligence in Predicting Clinical Outcome in COVID-19 Patients from Clinical, Biochemical and a Qualitative Chest X-Ray Scoring System. *RMI* **14**, 27–39 (2021).
49.
Ebrahimian, S. *et al.* Artificial intelligence matches subjective severity assessment of pneumonia for prediction of patient outcome and need for mechanical ventilation: a cohort study. *Sci Rep* **11**, 858 (2021).
50.
García Clemente, M. M. *et al.* Assessment of risk scores in Covid-19. *Int J Clin Pract* **75**, e13705 (2021).
51.
Cetinkal, G. *et al.* Assessment of the Modified CHA2DS2VASc Risk Score in Predicting Mortality in Patients Hospitalized With COVID-19. *American Journal of Cardiology* **135**, 143–149 (2020).
52.
Fang, X. *et al.* Association of AI quantified COVID-19 chest CT and patient outcome. *Int J Comput Assist Radiol Surg* **16**, 435–445 (2021).
53.
Sullivan, B. *et al.* Bayesian Prediction of Severe Outcomes in the LabMarCS: Laboratory Markers of COVID-19 Severity - Bristol Cohort. 2022.09.16.22279985 Preprint at https://doi.org/10.1101/2022.09.16.22279985 (2023).
54.

Kamran, S. M. *et al.* CALL Score and RAS Score as Predictive Models for Coronavirus Disease 2019. *Cureus* **12**, e11368 (2020).
55.
As, A. K. *et al.* Can a modified-simplified pulmonary embolism severity index (m-sPESI) be used to predict the need for intensive care in hospitalized COVID-19 patients? *J Thromb Thrombolysis* **52**, 759–765 (2021).
56.
Chen, Y. *et al.* CANPT Score: A Tool to Predict Severe COVID-19 on Admission. *Frontiers in Medicine* **8**, (2021).
57.
Zhou, S. *et al.* Chest CT imaging features and severity scores as biomarkers for prognostic prediction in patients with COVID-19. *Ann Transl Med* **8**, 1449 (2020).
58.
Francone, M. *et al.* Chest CT score in COVID-19 patients: correlation with disease severity and short-term prognosis. *Eur Radiol* **30**, 6808–6817 (2020).
59.
Halmaciu, I. *et al.* Chest CT Severity Score and Systemic Inflammatory Biomarkers as Predictors of the Need for Invasive Mechanical Ventilation and of COVID-19 Patients' Mortality. *Diagnostics (Basel)* **12**, 2089 (2022).
60.
Esposito, A. *et al.* Chest CT-derived pulmonary artery enlargement at the admission predicts overall survival in COVID-19 patients: insight from 1461 consecutive patients in Italy. *Eur Radiol* **31**, 4031–4041 (2021).
61.
Borghesi, A. *et al.* Chest X-ray severity index as a predictor of in-hospital mortality in coronavirus disease 2019: A study of 302 patients from Italy. *International Journal of Infectious Diseases* **96**, 291–293 (2020).
62.
Hoang, S. V. *et al.* Chest X-ray Severity Score as a Putative Predictor of Clinical Outcome in Hospitalized Patients: An Experience From a Vietnamese COVID-19 Field Hospital. *Cureus* **14**, e23323 (2022).
63.
Guan, X. *et al.* Clinical and inflammatory features based machine learning model for fatal risk prediction of hospitalized COVID-19 patients: results from a retrospective cohort study. *Ann Med* **53**, 257–266 (2021).
64.
Wang, K. *et al.* Clinical and Laboratory Predictors of In-hospital Mortality in Patients With Coronavirus Disease-2019: A Cohort Study in Wuhan, China. *Clin Infect Dis* **71**, 2079–2088 (2020).
65.
Yang, Q. *et al.* Clinical characteristics and a decision tree model to predict death outcome in severe COVID-19 patients. *BMC Infect Dis* **21**, 783 (2021).
66.
Yadaw, A. S. *et al.* Clinical features of COVID-19 mortality: development and validation of a clinical prediction model. *Lancet Digit Health* **2**, e516–e525 (2020).

67.
Kocadagli, O., Baygul, A., Gokmen, N., Incir, S. & Aktan, C. Clinical prognosis evaluation of COVID-19 patients: An interpretable hybrid machine learning approach. *Curr Res Transl Med* **70**, 103319 (2022).
68.
Al Hassan, H., Cocks, E., Jesani, L., Lewis, S. & Szakmany, T. Clinical Risk Prediction Scores in Coronavirus Disease 2019: Beware of Low Validity and Clinical Utility. *Crit Care Explor* **2**, e0253 (2020).
69.
Ageno, W. *et al.* Clinical risk scores for the early prediction of severe outcomes in patients hospitalized for COVID-19. *Intern Emerg Med* **16**, 989–996 (2021).
70.
Gavelli, F. *et al.* Clinical stability and in-hospital mortality prediction in COVID-19 patients presenting to the Emergency Department. *Minerva Med* **112**, 118–123 (2021).
71.
Hu, J. *et al.* Combination of serum lactate dehydrogenase and sex is predictive of severe disease in patients with COVID-19. *Medicine (Baltimore)* **99**, e22774 (2020).
72.
Yue, T. *et al.* Combined clinical and imaging features better predict the critical outcomes of patients with SARS-COV-2. *Medicine (Baltimore)* **100**, e25083 (2021).
73.
Kwon, Y. J. F. *et al.* Combining Initial Radiographs and Clinical Variables Improves Deep Learning Prognostication in Patients with COVID-19 from the Emergency Department. *Radiol Artif Intell* **3**, e200098 (2021).
74.
Xiong, Y. *et al.* Comparing different machine learning techniques for predicting COVID-19 severity. *Infect Dis Poverty* **11**, 19 (2022).
75.
Ak, R., Kurt, E. & Bahadirli, S. Comparison of 2 Risk Prediction Models Specific for COVID-19: The Brescia-COVID Respiratory Severity Scale Versus the Quick COVID-19 Severity Index. *Disaster Medicine and Public Health Preparedness* **15**, e46–e50 (2021).
76.
Abdulaal, A. *et al.* Comparison of deep learning with regression analysis in creating predictive models for SARS-CoV-2 outcomes. *BMC Medical Informatics and Decision Making* **20**, 299 (2020).
77.
Arru, C. *et al.* Comparison of deep learning, radiomics and subjective assessment of chest CT findings in SARS-CoV-2 pneumonia. *Clinical Imaging* **80**, 58–66 (2021).
78.
Shanbehzadeh, M. *et al.* Comparison of Machine Learning Tools for the Prediction of ICU Admission in COVID-19 Hospitalized Patients. *Shiraz E-Med J* **23**, (2022).
79.
Kibar Akilli, I. *et al.* Comparison of Pneumonia Severity Indices, qCSI, 4C-Mortality Score and qSOFA in Predicting Mortality in Hospitalized Patients with COVID-19 Pneumonia. *J Pers Med* **12**, 801 (2022).
80.

Homayounieh, F. *et al.* Computed Tomography Radiomics Can Predict Disease Severity and Outcome in Coronavirus Disease 2019 Pneumonia. *J Comput Assist Tomogr* **44**, 640–646 (2020).
81.
Cavallaro, M., Moiz, H., Keeling, M. J. & McCarthy, N. D. Contrasting factors associated with COVID-19-related ICU admission and death outcomes in hospitalised patients by means of Shapley values. *PLOS Computational Biology* **17**, e1009121 (2021).
82.
Bertsimas, D. *et al.* COVID-19 mortality risk assessment: An international multi-center study. *PLOS ONE* **15**, e0243262 (2020).
83.
Peacock, S. *et al.* COVID-19 Patient Outcome Prediction Using Selected Features from Emergency Department Data and Feed-Forward Neural Networks. in *Wireless Mobile Communication and Healthcare* (eds. Ye, J., O'Grady, M. J., Civitarese, G. & Yordanova, K.) 323–335 (Springer International Publishing, Cham, 2021). doi:10.1007/978-3-030-70569-5_21.
84.
Raad, M. *et al.* COVID-19 risk index (CRI): a simple and validated emergency department risk score that predicts mortality and the need for mechanical ventilation. *J Thromb Thrombolysis* **53**, 567–575 (2022).
85.
Garcia-Gordillo, J. A. *et al.* COVID-IRS: A novel predictive score for risk of invasive mechanical ventilation in patients with COVID-19. *PLoS One* **16**, e0248357 (2021).
86.
Nagy, Á., Ligeti, B., Szebeni, J., Pongor, S. & Gyrffy, B. COVIDOUTCOME-estimating COVID severity based on mutation signatures in the SARS-CoV-2 genome. *Database (Oxford)* **2021**, baab020 (2021).
87.
Abbasi, W. A., Abbas, S. A. & Andleeb, S. COVIDX: Computer-aided diagnosis of Covid-19 and its severity prediction with raw digital chest X-ray images. *Quant. Biol.* **10**, 208–220 (2022).
88.
Cai, W. *et al.* CT Quantification and Machine-learning Models for Assessment of Disease Severity and Prognosis of COVID-19 Patients. *Academic Radiology* **27**, 1665–1678 (2020).
89.
Homayounieh, F. *et al.* CT Radiomics, Radiologists, and Clinical Information in Predicting Outcome of Patients with COVID-19 Pneumonia. *Radiol Cardiothorac Imaging* **2**, e200322 (2020).
90.
Alrajhi, A. A. *et al.* Data-Driven Prediction for COVID-19 Severity in Hospitalized Patients. *International Journal of Environmental Research and Public Health* **19**, 2958 (2022).
91.
Fang, C. *et al.* Deep learning for predicting COVID-19 malignant progression. *Med Image Anal* **72**, 102096 (2021).
92.
Ho, T. T. *et al.* Deep Learning Models for Predicting Severe Progression in COVID-19-Infected Patients: Retrospective Study. *JMIR Med Inform* **9**, e24973 (2021).
93.

Li, X. *et al.* Deep learning prediction of likelihood of ICU admission and mortality in COVID-19 patients using clinical variables. *PeerJ* **8**, e10337 (2020).
94.
Bermejo-Peláez, D. *et al.* Deep learning-based lesion subtyping and prediction of clinical outcomes in COVID-19 pneumonia using chest CT. *Sci Rep* **12**, 9387 (2022).
95.
Zhu, J. S. *et al.* Deep-learning artificial intelligence analysis of clinical variables predicts mortality in COVID-19 patients. *J Am Coll Emerg Physicians Open* **1**, 1364–1373 (2020).
96.
Mann, C. Z. *et al.* Derivation and external validation of a simple risk score to predict in-hospital mortality in patients hospitalized for COVID-19: A multicenter retrospective cohort study. *Medicine (Baltimore)* **100**, e27422 (2021).
97.
Gerotziafas, G. T. *et al.* Derivation and Validation of a Predictive Score for Disease Worsening in Patients with COVID-19. *Thromb Haemost* **120**, 1680–1690 (2020).
98.
Li, J. *et al.* Derivation and validation of a prognostic model for predicting in-hospital mortality in patients admitted with COVID-19 in Wuhan, China: the PLANS (platelet lymphocyte age neutrophil sex) model. *BMC Infect Dis* **20**, 959 (2020).
99.
Foieni, F. *et al.* Derivation and validation of the clinical prediction model for COVID-19. *Intern Emerg Med* **15**, 1409–1414 (2020).
100.
Levine, D. M. *et al.* Derivation of a Clinical Risk Score to Predict 14-Day Occurrence of Hypoxia, ICU Admission, and Death Among Patients with Coronavirus Disease 2019. *J Gen Intern Med* **36**, 730–737 (2021).
101.
Shanbehzadeh, M., Nopour, R. & Kazemi-Arpanahi, H. Design of an artificial neural network to predict mortality among COVID-19 patients. *Inform Med Unlocked* **31**, 100983 (2022).
102.
Jimenez-Solem, E. *et al.* Developing and validating COVID-19 adverse outcome risk prediction models from a bi-national European cohort of 5594 patients. *Sci Rep* **11**, 3246 (2021).
103.
Li, S. *et al.* Development and external evaluation of predictions models for mortality of COVID-19 patients using machine learning method. *Neural Comput Appl* **35**, 13037–13046 (2023).
104.
Hajifathalian, K. *et al.* Development and external validation of a prediction risk model for short-term mortality among hospitalized U.S. COVID-19 patients: A proposal for the COVID-AID risk tool. *PLoS One* **15**, e0239536 (2020).
105.
Xie, J. *et al.* Development and external validation of a prognostic multivariable model on admission for hospitalized patients with COVID-19. 2020.03.28.20045997 Preprint at https://doi.org/10.1101/2020.03.28.20045997 (2020).
106.

Chow, D. S. *et al.* Development and external validation of a prognostic tool for COVID-19 critical disease. *PLOS ONE* **15**, e0242953 (2020).
107.
Adderley, N. J. *et al.* Development and external validation of prognostic models for COVID-19 to support risk stratification in secondary care. *BMJ Open* **12**, e049506 (2022).
108.
Li, L. *et al.* Development and multicenter validation of a CT-based radiomics signature for predicting severe COVID-19 pneumonia. *Eur Radiol* **31**, 7901–7912 (2021).
109.
Zhou, Y. *et al.* Development and validation a nomogram for predicting the risk of severe COVID-19: A multi-center study in Sichuan, China. *PLoS One* **15**, e0233328 (2020).
110.
Gude, F. *et al.* Development and validation of a clinical score to estimate progression to severe or critical state in COVID-19 pneumonia hospitalized patients. *Sci Rep* **10**, 19794 (2020).
111.
Xiao, L. *et al.* Development and Validation of a Deep Learning-Based Model Using Computed Tomography Imaging for Predicting Disease Severity of Coronavirus Disease 2019. *Front Bioeng Biotechnol* **8**, 898 (2020).
112.
Quiroz, J. C. *et al.* Development and Validation of a Machine Learning Approach for Automated Severity Assessment of COVID-19 Based on Clinical and Imaging Data: Retrospective Study. *JMIR Med Inform* **9**, e24572 (2021).
113.
Gupta, A. *et al.* Development and Validation of a Multivariable Risk Prediction Model for COVID-19 Mortality in the Southern United States. *Mayo Clin Proc* **96**, 3030–3041 (2021).
114.
Ma, K. *et al.* Development and Validation of a New Prognostic Scoring System for COVID-19. *Jpn J Infect Dis* **74**, 359–366 (2021).
115.
Li, X. *et al.* Development and validation of a nomogram for predicting the disease progression of nonsevere coronavirus disease 2019. *Journal of Translational Internal Medicine* **9**, 131–142 (2021).
116.
Bartoletti, M. *et al.* Development and validation of a prediction model for severe respiratory failure in hospitalized patients with SARS-CoV-2 infection: a multicentre cohort study (PREDI-CO study). *Clinical Microbiology and Infection* **26**, 1545–1553 (2020).
117.
Meng, Z. *et al.* Development and Validation of a Predictive Model for Severe COVID-19: A Case-Control Study in China. *Front Med (Lausanne)* **8**, 663145 (2021).
118.
Schöning, V. *et al.* Development and validation of a prognostic COVID-19 severity assessment (COSA) score and machine learning models for patient triage at a tertiary hospital. *J Transl Med* **19**, 56 (2021).
119.
Gude-Sampedro, F. *et al.* Development and validation of a prognostic model based on comorbidities to predict COVID-19 severity: a population-based study. *Int J Epidemiol* **50**, 64–74 (2021).

120.
Li, L. *et al.* Development and validation of a prognostic nomogram for predicting in-hospital mortality of COVID-19: a multicenter retrospective cohort study of 4086 cases in China. *Aging (Albany NY)* **13**, 3176–3189 (2021).
121.
Yuan, Y. *et al.* Development and Validation of a Prognostic Risk Score System for COVID-19 Inpatients: A Multi-Center Retrospective Study in China. *Engineering (Beijing)* **8**, 116–121 (2022).
122.
Zhang, S. *et al.* Development and validation of a risk factor-based system to predict short-term survival in adult hospitalized patients with COVID-19: a multicenter, retrospective, cohort study. *Crit Care* **24**, 438 (2020).
123.
Woo, S. H. *et al.* Development and Validation of a Web-Based Severe COVID-19 Risk Prediction Model. *Am J Med Sci* **362**, 355–362 (2021).
124.
Rahman, T. *et al.* Development and Validation of an Early Scoring System for Prediction of Disease Severity in COVID-19 Using Complete Blood Count Parameters. *IEEE Access* **9**, 120422–120441 (2021).
125.
Smit, J. M. *et al.* Development and validation of an early warning model for hospitalized COVID-19 patients: a multi-center retrospective cohort study. *Intensive Care Med Exp* **10**, 38 (2022).
126.
Gao, Y. *et al.* Development and validation of an online model to predict critical COVID-19 with immune-inflammatory parameters. *Journal of Intensive Care* **9**, 19 (2021).
127.
Hao, B. *et al.* Development and validation of predictive models for COVID-19 outcomes in a safety-net hospital population. *J Am Med Inform Assoc* **29**, 1253–1262 (2022).
128.
Ma, X. *et al.* Development and validation of prognosis model of mortality risk in patients with COVID-19. *Epidemiol Infect* **148**, e168 (2020).
129.
Mei, Q. *et al.* Development and validation of prognostic model for predicting mortality of COVID-19 patients in Wuhan, China. *Sci Rep* **10**, 22451 (2020).
130.
Shankar, V. *et al.* Development and validation of prognostic scoring system for COVID-19 severity in South India. *Ir J Med Sci* **191**, 2823–2831 (2022).
131.
Gupta, R. K. *et al.* Development and validation of the ISARIC 4C Deterioration model for adults hospitalised with COVID-19: a prospective cohort study. *Lancet Respir Med* **9**, 349–359 (2021).
132.
Alvarez-Uria, G. *et al.* Development and Validation of the RCOS Prognostic Index: A Bedside Multivariable Logistic Regression Model to Predict Hypoxaemia or Death in Patients with SARS-CoV-2 Infection. *Interdisciplinary Perspectives on Infectious Diseases* **2022**, e2360478 (2022).
133.

Wu, G. *et al.* Development of a clinical decision support system for severity risk prediction and triage of COVID-19 patients at hospital admission: an international multicentre study. *Eur Respir J* **56**, 2001104 (2020).
134.
Boss, A. N. *et al.* Development of a Mortality Prediction Model in Hospitalised SARS-CoV-2 Positive Patients Based on Routine Kidney Biomarkers. *International Journal of Molecular Sciences* **23**, 7260 (2022).
135.
Zhou, J. *et al.* Development of a multivariable prediction model for severe COVID-19 disease: a population-based study from Hong Kong. *NPJ Digit Med* **4**, 66 (2021).
136.
Allenbach, Y. *et al.* Development of a multivariate prediction model of intensive care unit transfer or death: A French prospective cohort study of hospitalized COVID-19 patients. *PLOS ONE* **15**, e0240711 (2020).
137.
Bellos, I. *et al.* Development of a novel risk score for the prediction of critical illness amongst COVID-19 patients. *International Journal of Clinical Practice* **75**, e13915 (2021).
138.
Booth, A. L., Abels, E. & McCaffrey, P. Development of a prognostic model for mortality in COVID-19 infection using machine learning. *Modern Pathology* **34**, 522–531 (2021).
139.
Fukushima, K. *et al.* Development of a Risk Prediction Score to Identify High-Risk Groups for the Critical Coronavirus Disease 2019 (COVID-19) in Japan. *Jpn J Infect Dis* **74**, 344–351 (2021).
140.
Marcos, M. *et al.* Development of a severity of disease score and classification model by machine learning for hospitalized COVID-19 patients. *PLoS One* **16**, e0240200 (2021).
141.
Wang, H. *et al.* Development of an Early Warning Model for Predicting the Death Risk of Coronavirus Disease 2019 Based on Data Immediately Available on Admission. *Front Med (Lausanne)* **8**, 699243 (2021).
142.
Subhani, F. *et al.* Development of COVID-19 severity assessment score in adults presenting with COVID-19 to the emergency department. *BMC Infectious Diseases* **22**, 576 (2022).
143.
Sarkar, A. *et al.* Development of lab score system for predicting COVID-19 patient severity: A retrospective analysis. *PLoS One* **17**, e0273006 (2022).
144.
Wongvibulsin, S. *et al.* Development of Severe COVID-19 Adaptive Risk Predictor (SCARP), a Calculator to Predict Severe Disease or Death in Hospitalized Patients With COVID-19. *Ann Intern Med* **174**, 777–785 (2021).
145.
Calvillo-Batllés, P. *et al.* Development of severity and mortality prediction models for covid-19 patients at emergency department including the chest x-ray. *Radiología (English Edition)* **64**, 214–227 (2022).
146.

Gao, Y. *et al.* Diagnostic utility of clinical laboratory data determinations for patients with the severe COVID-19. *J Med Virol* **92**, 791–796 (2020).
147.
Hu, H. *et al.* Early prediction and identification for severe patients during the pandemic of COVID-19: A severe COVID-19 risk model constructed by multivariate logistic regression analysis. *J Glob Health* **10**, 020510 (2020).
148.
Liu, L. *et al.* Early prediction model for progression and prognosis of severe patients with coronavirus disease 2019. *Medicine (Baltimore)* **100**, e24901 (2021).
149.
Richard, V. R. *et al.* Early Prediction of COVID-19 Patient Survival by Targeted Plasma Multi-Omics and Machine Learning. *Mol Cell Proteomics* **21**, 100277 (2022).
150.
Aljouie, A. F. *et al.* Early Prediction of COVID-19 Ventilation Requirement and Mortality from Routinely Collected Baseline Chest Radiographs, Laboratory, and Clinical Data with Machine Learning. *JMDH* **14**, 2017–2033 (2021).
151.
Feng, Z. *et al.* Early prediction of disease progression in COVID-19 pneumonia patients with chest CT and clinical characteristics. *Nat Commun* **11**, 4968 (2020).
152.
Garrafa, E. *et al.* Early prediction of in-hospital death of COVID-19 patients: a machine-learning model based on age, blood analyses, and chest x-ray score. *Elife* **10**, e70640 (2021).
153.
Hao, B. *et al.* Early prediction of level-of-care requirements in patients with COVID-19. *Elife* **9**, e60519 (2020).
154.
Hu, C. *et al.* Early prediction of mortality risk among patients with severe COVID-19, using machine learning. *Int J Epidemiol* **49**, 1918–1929 (2021).
155.
Mustafić, S. *et al.* Early predictors of severity and mortality in COVID-19 hospitalized patients. *Med Glas (Zenica)* **18**, 384–393 (2021).
156.
Chua, F. *et al.* Early prognostication of COVID-19 to guide hospitalisation versus outpatient monitoring using a point-of-test risk prediction score. *Thorax* **76**, 696–703 (2021).
157.
Liang, W. *et al.* Early triage of critically ill COVID-19 patients using deep learning. *Nat Commun* **11**, 3543 (2020).
158.
Ortiz, A. *et al.* Effective deep learning approaches for predicting COVID-19 outcomes from chest computed tomography volumes. *Sci Rep* **12**, 1716 (2022).
159.
Nicholson, C. J. *et al.* Estimating risk of mechanical ventilation and in-hospital mortality among adult COVID-19 patients admitted to Mass General Brigham: The VICE and DICE scores. *EClinicalMedicine* **33**, 100765 (2021).

160.
Dong, Y. *et al.* Evaluating the ability of the NLHA2 and artificial neural network models to predict COVID-19 severity, and comparing them with the four existing scoring systems. *Microb Pathog* **171**, 105735 (2022).
161.
Carr, E. *et al.* Evaluation and improvement of the National Early Warning Score (NEWS2) for COVID-19: a multi-hospital study. *BMC Medicine* **19**, 23 (2021).
162.
Matos, J. *et al.* Evaluation of novel coronavirus disease (COVID-19) using quantitative lung CT and clinical data: prediction of short-term outcome. *Eur Radiol Exp* **4**, 39 (2020).
163.
Gülbay, M. *et al.* Evaluation of the models generated from clinical features and deep learning-based segmentations: Can thoracic CT on admission help us to predict hospitalized COVID-19 patients who will require intensive care? *BMC Med Imaging* **22**, 110 (2022).
164.
Casiraghi, E. *et al.* Explainable Machine Learning for Early Assessment of COVID-19 Risk Prediction in Emergency Departments. *IEEE Access* **8**, 196299–196325 (2020).
165.
Paraskevas, T. *et al.* External validation of the 4C Mortality Score and PRIEST COVID-19 Clinical Severity Score in patients hospitalized with COVID-19 pneumonia in Greece. *Rom J Intern Med* **60**, 244–249 (2022).
166.
Ramón, A. *et al.* eXtreme Gradient Boosting-based method to classify patients with COVID-19. *J Investig Med* jim-2021-002278 (2022) doi:10.1136/jim-2021-002278.
167.
Faria, S. P. *et al.* Forecasting COVID-19 Severity by Intelligent Optical Fingerprinting of Blood Samples. *Diagnostics (Basel)* **11**, 1309 (2021).
168.
Crowley, G., Kwon, S., Mengling, L. & Nolan, A. ICU Admission and Mortality Prediction in Severe COVID-19: A Machine Learning Approach. in *TP49. TP049 COVID: ARDS AND ICU MANAGEMENT* A2564–A2564 (American Thoracic Society, 2021). doi:10.1164/ajrccm-conference.2021.203.1_MeetingAbstracts.A2564.
169.
Wu, S. *et al.* Identification and Validation of a Novel Clinical Signature to Predict the Prognosis in Confirmed Coronavirus Disease 2019 Patients. *Clin Infect Dis* **71**, 3154–3162 (2020).
170.
Di, B. *et al.* Identification and validation of predictive factors for progression to severe COVID-19 pneumonia by proteomics. *Signal Transduct Target Ther* **5**, 217 (2020).
171.
Zhang, S. *et al.* Identification and validation of prognostic factors in patients with COVID-19: A retrospective study based on artificial intelligence algorithms. *J Intensive Med* **1**, 103–109 (2021).
172.
Laguna-Goya, R. *et al.* IL-6-based mortality risk model for hospitalized patients with COVID-19. *J Allergy Clin Immunol* **146**, 799-807.e9 (2020).

173.
Besutti, G. *et al.* Imaging-based indices combining disease severity and time from disease onset to predict COVID-19 mortality: A cohort study. *PLOS ONE* **17**, e0270111 (2022).
174.
Huang, J., Cheng, A., Lin, S., Zhu, Y. & Chen, G. Individualized prediction nomograms for disease progression in mild COVID-19. *J Med Virol* **92**, 2074–2080 (2020).
175.
Hiremath, A. *et al.* Integrated Clinical and CT Based Artificial Intelligence Nomogram for Predicting Severity and Need for Ventilator Support in COVID-19 Patients: A Multi-Site Study. *IEEE J Biomed Health Inform* **25**, 4110–4118 (2021).
176.
Lassau, N. *et al.* Integrating deep learning CT-scan model, biological and clinical variables to predict severity of COVID-19 patients. *Nat Commun* **12**, 634 (2021).
177.
O'Shea, A. *et al.* Intubation and mortality prediction in hospitalized COVID-19 patients using a combination of convolutional neural network-based scoring of chest radiographs and clinical data. *BJR Open* **4**, 20210062 (2022).
178.
Clift, A. K. *et al.* Living risk prediction algorithm (QCOVID) for risk of hospital admission and mortality from coronavirus 19 in adults: national derivation and validation cohort study. *BMJ* **371**, m3731 (2020).
179.
Ye, J., Hua, M. & Zhu, F. Machine Learning Algorithms are Superior to Conventional Regression Models in Predicting Risk Stratification of COVID-19 Patients. *Risk Manag Healthc Policy* **14**, 3159–3166 (2021).
180.
Passarelli-Araujo, H., Passarelli-Araujo, H., Urbano, M. R. & Pescim, R. R. Machine learning and comorbidity network analysis for hospitalized patients with COVID-19 in a city in Southern Brazil. *Smart Health (Amst)* **26**, 100323 (2022).
181.
Aktar, S. *et al.* Machine Learning Approach to Predicting COVID-19 Disease Severity Based on Clinical Blood Test Data: Statistical Analysis and Model Development. *JMIR Medical Informatics* **9**, e25884 (2021).
182.
Laatifi, M. *et al.* Machine learning approaches in Covid-19 severity risk prediction in Morocco. *J Big Data* **9**, 5 (2022).
183.
Gao, Y. *et al.* Machine learning based early warning system enables accurate mortality risk prediction for COVID-19. *Nat Commun* **11**, 5033 (2020).
184.
Feng, Z. *et al.* Machine learning based on clinical characteristics and chest CT quantitative measurements for prediction of adverse clinical outcomes in hospitalized patients with COVID-19. *Eur Radiol* **31**, 7925–7935 (2021).
185.

Patel, D. *et al.* Machine learning based predictors for COVID-19 disease severity. *Sci Rep* **11**, 4673 (2021).
186.
Ferrari, D. *et al.* Machine learning in predicting respiratory failure in patients with COVID-19 pneumonia-Challenges, strengths, and opportunities in a global health emergency. *PLoS One* **15**, e0239172 (2020).
187.
Reina Reina, A. *et al.* Machine learning model from a Spanish cohort for prediction of SARS-COV-2 mortality risk and critical patients. *Sci Rep* **12**, 5723 (2022).
188.
Kang, J. *et al.* Machine learning predictive model for severe COVID-19. *Infection, Genetics and Evolution* **90**, 104737 (2021).
189.
Purkayastha, S. *et al.* Machine Learning-Based Prediction of COVID-19 Severity and Progression to Critical Illness Using CT Imaging and Clinical Data. *Korean J Radiol* **22**, 1213–1224 (2021).
190.
Banoei, M. M., Dinparastisaleh, R., Zadeh, A. V. & Mirsaeidi, M. Machine-learning-based COVID-19 mortality prediction model and identification of patients at low and high risk of dying. *Critical Care* **25**, 328 (2021).
191.
González-Cebrián, A. *et al.* Machine-learning-derived predictive score for early estimation of COVID-19 mortality risk in hospitalized patients. *PLoS One* **17**, e0274171 (2022).
192.
McRae, M. P. *et al.* Managing COVID-19 With a Clinical Decision Support Tool in a Community Health Network: Algorithm Development and Validation. *J Med Internet Res* **22**, e22033 (2020).
193.
Schalekamp, S. *et al.* Model-based Prediction of Critical Illness in Hospitalized Patients with COVID-19. *Radiology* **298**, E46–E54 (2021).
194.
Homayounieh, F. *et al.* Multicenter Assessment of CT Pneumonia Analysis Prototype for Predicting Disease Severity and Patient Outcome. *J Digit Imaging* **34**, 320–329 (2021).
195.
Myrstad, M. *et al.* National Early Warning Score 2 (NEWS2) on admission predicts severe disease and in-hospital mortality from Covid-19 - a prospective cohort study. *Scand J Trauma Resusc Emerg Med* **28**, 66 (2020).
196.
Fan, T. *et al.* Nomogram for Predicting COVID-19 Disease Progression Based on Single-Center Data: Observational Study and Model Development. *JMIR Med Inform* **8**, e19588 (2020).
197.
Tang, W. *et al.* Nomogram prediction of severe risk in patients with COVID-19 pneumonia. *Epidemiol Infect* **149**, e251 (2021).
198.
Surme, S. *et al.* Novel biomarker-based score (SAD-60) for predicting mortality in patients with COVID-19 pneumonia: a multicenter retrospective cohort of 1013 patients. *Biomark Med* **16**, 577–588 (2022).

199.
Keri, V. C. *et al.* Novel Scoring Systems to Predict the Need for Oxygenation and ICU Care, and Mortality in Hospitalized COVID-19 Patients: A Risk Stratification Tool. *Cureus* **14**, e27459 (2022).
200.
Schmidt, W., Jóźwiak, B., Czabajska, Z., Pawlak-Buś, K. & Leszczynski, P. On-admission laboratory predictors for developing critical COVID-19 during hospitalization - a multivariable logistic regression model. *Ann Agric Environ Med* **29**, 274–280 (2022).
201.
Ning, W. *et al.* Open resource of clinical data from patients with pneumonia for the prediction of COVID-19 outcomes via deep learning. *Nat Biomed Eng* **4**, 1197–1207 (2020).
202.
Kim, J. *et al.* Optimal Triage for COVID-19 Patients Under Limited Health Care Resources With a Parsimonious Machine Learning Prediction Model and Threshold Optimization Using Discrete-Event Simulation: Development Study. *JMIR Med Inform* **9**, e32726 (2021).
203.
Satici, C. *et al.* Performance of pneumonia severity index and CURB-65 in predicting 30-day mortality in patients with COVID-19. *Int J Infect Dis* **98**, 84–89 (2020).
204.
van Dam, P. M. E. L. *et al.* Performance of prediction models for short-term outcome in COVID-19 patients in the emergency department: a retrospective study. *Ann Med* **53**, 402–409 (2021).
205.
Doğanay, F. & Ak, R. Performance of the CURB-65, ISARIC-4C and COVID-GRAM scores in terms of severity for COVID-19 patients. *Int J Clin Pract* **75**, e14759 (2021).
206.
Rodriguez-Nava, G. *et al.* Performance of the quick COVID-19 severity index and the Brescia-COVID respiratory severity scale in hospitalized patients with COVID-19 in a community hospital setting. *Int J Infect Dis* **102**, 571–576 (2021).
207.
Wollenstein-Betech, S., Cassandras, C. G. & Paschalidis, I. Ch. Personalized Predictive Models for Symptomatic COVID-19 Patients Using Basic Preconditions. *medRxiv* 2020.05.03.20089813 (2020) doi:10.1101/2020.05.03.20089813.
208.
Wollenstein-Betech, S., Silva, A. A. B., Fleck, J. L., Cassandras, C. G. & Paschalidis, I. Ch. Physiological and socioeconomic characteristics predict COVID-19 mortality and resource utilization in Brazil. *PLoS One* **15**, e0240346 (2020).
209.
Cheng, P. *et al.* Pneumonia scoring systems for severe COVID-19: which one is better. *Virology Journal* **18**, 33 (2021).
210.
Bae, J. *et al.* Predicting Mechanical Ventilation and Mortality in COVID-19 Using Radiomics and Deep Learning on Chest Radiographs: A Multi-Institutional Study. *Diagnostics* **11**, 1812 (2021).
211.

Bello-Chavolla, O. Y. *et al.* Predicting Mortality Due to SARS-CoV-2: A Mechanistic Score Relating Obesity and Diabetes to COVID-19 Outcomes in Mexico. *The Journal of Clinical Endocrinology & Metabolism* **105**, 2752–2761 (2020).
212.
Tseng, L. *et al.* Predicting Poor Outcome of COVID-19 Patients on the Day of Admission with the COVID-19 Score. *Crit Care Res Pract* **2021**, 5585291 (2021).
213.
Campbell, T. W. *et al.* Predicting prognosis in COVID-19 patients using machine learning and readily available clinical data. *International Journal of Medical Informatics* **155**, 104594 (2021).
214.
Wang, M. *et al.* Predicting progression to severe COVID-19 using the PAINT score. *BMC Infect Dis* **22**, 498 (2022).
215.
Kurban, L. A. S. *et al.* Predicting Severe Disease and Critical Illness on Initial Diagnosis of COVID-19: Simple Triage Tools. *Front Med (Lausanne)* **9**, 817549 (2022).
216.
Chen, X. *et al.* Predicting severe or critical symptoms in hospitalized patients with COVID-19 from Yichang, China. *Aging* **13**, 1608–1619 (2020).
217.
Ryan, C. *et al.* Predicting severe outcomes in Covid-19 related illness using only patient demographics, comorbidities and symptoms. *Am J Emerg Med* **45**, 378–384 (2021).
218.
Salaffi, F. *et al.* Predicting Severe/Critical Outcomes in Patients With SARS-CoV2 Pneumonia: Development of the prediCtion seveRe/crItical ouTcome in COVID-19 (CRITIC) Model. *Front Med (Lausanne)* **8**, 695195 (2021).
219.
Ye, Y. *et al.* Prediction and follow-up of risk factors for severe SARS-CoV-2 pneumonia and application of CT visual scoring. *Technol Health Care* **29**, 153–164 (2021).
220.
Ji, D. *et al.* Prediction for Progression Risk in Patients With COVID-19 Pneumonia: The CALL Score. *Clin Infect Dis* **71**, 1393–1399 (2020).
221.
Luo, Y. *et al.* Prediction Model Based on the Combination of Cytokines and Lymphocyte Subsets for Prognosis of SARS-CoV-2 Infection. *J Clin Immunol* **40**, 960–969 (2020).
222.
Li, D. *et al.* Prediction of COVID-19 Severity Using Chest Computed Tomography and Laboratory Measurements: Evaluation Using a Machine Learning Approach. *JMIR Med Inform* **8**, e21604 (2020).
223.
Statsenko, Y., Al Zahmi, F., Habuza, T., Gorkom, K. N.-V. & Zaki, N. Prediction of COVID-19 severity using laboratory findings on admission: informative values, thresholds, ML model performance. *BMJ Open* **11**, e044500 (2021).
224.
Li, Y. *et al.* Prediction of disease progression in patients with COVID-19 by artificial intelligence assisted lesion quantification. *Sci Rep* **10**, 22083 (2020).

225.
Xu, F. *et al.* Prediction of Disease Progression of COVID-19 Based upon Machine Learning. *Int J Gen Med* **14**, 1589–1598 (2021).
226.
De Giorgi, A. *et al.* Prediction of in-hospital mortality of patients with SARS-CoV-2 infection by comorbidity indexes: an Italian internal medicine single center study. *Eur Rev Med Pharmacol Sci* **24**, 10258–10266 (2020).
227.
Altini, N. *et al.* Predictive Machine Learning Models and Survival Analysis for COVID-19 Prognosis Based on Hematochemical Parameters. *Sensors* **21**, 8503 (2021).
228.
Gómez, L. C., Curto, S. V., Sebastian, M. B. P., Jiménez, B. F. & Duniol, M. D. Predictive Model of Severity in SARS CoV-2 Patients at Hospital Admission Using Blood-Related Parameters. *EJIFCC* **32**, 255–264 (2021).
229.
Liu, S., Yao, N., Qiu, Y. & He, C. Predictive performance of SOFA and qSOFA for in-hospital mortality in severe novel coronavirus disease. *Am J Emerg Med* **38**, 2074–2080 (2020).
230.
Roul, P. K. *et al.* Predictive Value of Chest CT Score in Assessing Disease Severity and Short-term Mortality in COVID-19 Pneumonia at a Tertiary Care Centre in Northern India: A Prospective Observational Study. *JCDR* (2022) doi:10.7860/JCDR/2022/51808.16168.
231.
Wang, Z. *et al.* Predictive Value of Prognostic Nutritional Index on COVID-19 Severity. *Front Nutr* **7**, 582736 (2021).
232.
Khayat Kashani, H. R., Hajijafari, M., Khayat Kashani, F. & Salimi, S. Predictive value of the preliminary findings in the severity of COVID-19 disease and the effect on therapeutic approaches. *Dermatol Ther* **34**, e14828 (2021).
233.
Hu, X. *et al.* Predictive value of the prognostic nutritional index for the severity of coronavirus disease 2019. *Nutrition* **84**, 111123 (2021).
234.
Yitao, Z. *et al.* Predictors of clinical deterioration in non-severe patients with COVID-19: a retrospective cohort study. *Curr Med Res Opin* **37**, 385–391 (2021).
235.
Gopalan, N. *et al.* Predictors of mortality among hospitalized COVID-19 patients and risk score formulation for prioritizing tertiary care-An experience from South India. *PLoS One* **17**, e0263471 (2022).
236.
España, P. P. *et al.* Predictors of mortality of COVID-19 in the general population and nursing homes. *Intern Emerg Med* **16**, 1487–1496 (2021).
237.

Araújo, D. C., Veloso, A. A., Borges, K. B. G. & Carvalho, M. das G. Prognosing the risk of COVID-19 death through a machine learning-based routine blood panel: A retrospective study in Brazil. *International Journal of Medical Informatics* **165**, 104835 (2022).
238.
Nuevo-Ortega, P. *et al.* Prognosis of COVID-19 pneumonia can be early predicted combining Age-adjusted Charlson Comorbidity Index, CRB score and baseline oxygen saturation. *Sci Rep* **12**, 2367 (2022).
239.
Cho, S.-Y. *et al.* Prognosis Score System to Predict Survival for COVID-19 Cases: a Korean Nationwide Cohort Study. *Journal of Medical Internet Research* **23**, e26257 (2021).
240.
Jang, J. G., Hur, J., Hong, K. S., Lee, W. & Ahn, J. H. Prognostic Accuracy of the SIRS, qSOFA, and NEWS for Early Detection of Clinical Deterioration in SARS-CoV-2 Infected Patients. *Journal of Korean Medical Science* **35**, (2020).
241.
Huang, H. *et al.* Prognostic Factors for COVID-19 Pneumonia Progression to Severe Symptoms Based on Earlier Clinical Features: A Retrospective Analysis. *Front Med (Lausanne)* **7**, 557453 (2020).
242.
Abdulaal, A. *et al.* Prognostic Modeling of COVID-19 Using Artificial Intelligence in the United Kingdom: Model Development and Validation. *Journal of Medical Internet Research* **22**, e20259 (2020).
243.
Jiao, Z. *et al.* Prognostication of patients with COVID-19 using artificial intelligence based on chest x-rays and clinical data: a retrospective study. *The Lancet Digital Health* **3**, e286–e294 (2021).
244.
Vultaggio, A. *et al.* Prompt Predicting of Early Clinical Deterioration of Moderate-to-Severe COVID-19 Patients: Usefulness of a Combined Score Using IL-6 in a Preliminary Study. *J Allergy Clin Immunol Pract* **8**, 2575-2581.e2 (2020).
245.
Carbonell, G. *et al.* Quantitative chest CT combined with plasma cytokines predict outcomes in COVID-19 patients. 2021.10.11.21264709 Preprint at https://doi.org/10.1101/2021.10.11.21264709 (2021).
246.
Ke, Z. *et al.* Radiomics analysis enables fatal outcome prediction for hospitalized patients with coronavirus disease 2019 (COVID-19). *Acta Radiol* **63**, 319–327 (2022).
247.
Kim, K. M. *et al.* Rapid prediction of in-hospital mortality among adults with COVID-19 disease. *PLoS One* **17**, e0269813 (2022).
248.
Wickstrøm, K. E. *et al.* Regional performance variation in external validation of four prediction models for severity of COVID-19 at hospital admission: An observational multi-centre cohort study. *PLoS One* **16**, e0255748 (2021).
249.
Ghani, H. *et al.* Relevance of prediction scores derived from the SARS-CoV-2 first wave, in the evolving UK COVID-19 second wave, for safe early discharge and mortality: a PREDICT COVID-19 UK prospective observational cohort study. *BMJ Open* **12**, e054469 (2022).

250.
Polilli, E. *et al.* Reliability of predictive models to support early decision making in the emergency department for patients with confirmed diagnosis of COVID-19: the Pescara Covid Hospital score. *BMC Health Serv Res* **22**, 1062 (2022).
251.
Nieto-Codesido, I. *et al.* Risk Factors of Mortality in Hospitalized Patients With COVID-19 Applying a Machine Learning Algorithm. *Open Respir Arch* **4**, 100162 (2022).
252.
Zhang, H. *et al.* Risk prediction for poor outcome and death in hospital in-patients with COVID-19: derivation in Wuhan, China and external validation in London, UK. 2020.04.28.20082222 Preprint at https://doi.org/10.1101/2020.04.28.20082222 (2020).
253.
Knight, S. R. *et al.* Risk stratification of patients admitted to hospital with covid-19 using the ISARIC WHO Clinical Characterisation Protocol: development and validation of the 4C Mortality Score. *BMJ* **370**, m3339 (2020).
254.
Baikpour, M. *et al.* Role of a Chest X-ray Severity Score in a Multivariable Predictive Model for Mortality in Patients with COVID-19: A Single-Center, Retrospective Study. *Journal of Clinical Medicine* **11**, 2157 (2022).
255.
Gianstefani, A. *et al.* Role of ROX index in the first assessment of COVID-19 patients in the emergency department. *Intern Emerg Med* **16**, 1959–1965 (2021).
256.
Dashti, H., Roche, E. C., Bates, D. W., Mora, S. & Demler, O. SARS2 simplified scores to estimate risk of hospitalization and death among patients with COVID-19. 2020.09.11.20190520 Preprint at https://doi.org/10.1101/2020.09.11.20190520 (2020).
257.
Shang, Y. *et al.* Scoring systems for predicting mortality for severe patients with COVID-19. *EClinicalMedicine* **24**, 100426 (2020).
258.
Chang, Y. *et al.* Severe versus common COVID-19: an early warning nomogram model. *Aging* **14**, 544–556 (2022).
259.
Lee, J., Ta, C., Kim, J. H., Liu, C. & Weng, C. Severity Prediction for COVID-19 Patients via Recurrent Neural Networks. *medRxiv* 2020.08.28.20184200 (2021) doi:10.1101/2020.08.28.20184200.
260.
Artero, A. *et al.* Severity Scores in COVID-19 Pneumonia: a Multicenter, Retrospective, Cohort Study. *J GEN INTERN MED* **36**, 1338–1345 (2021).
261.
Riveiro-Barciela, M. *et al.* Simple predictive models identify patients with COVID-19 pneumonia and poor prognosis. *PLoS One* **15**, e0244627 (2020).
262.
Zhao, Y. *et al.* Statistical Analysis and Machine Learning Prediction of Disease Outcomes for COVID-19 and Pneumonia Patients. *Front Cell Infect Microbiol* **12**, 838749 (2022).

263.
Ilbeigipour, S. & Albadvi, A. Supervised learning of COVID-19 patients' characteristics to discover symptom patterns and improve patient outcome prediction. *Inform Med Unlocked* **30**, 100933 (2022).
264.
Jamshidi, E. *et al.* Symptom Prediction and Mortality Risk Calculation for COVID-19 Using Machine Learning. *Front Artif Intell* **4**, 673527 (2021).
265.
Kaeuffer, C. *et al.* The BAS2IC Score: A Useful Tool to Identify Patients at High Risk of Early Progression to Severe Coronavirus Disease 2019. *Open Forum Infect Dis* **7**, ofaa405 (2020).
266.
Hohl, C. M. *et al.* The CCEDRRN COVID-19 Mortality Score to predict death among nonpalliative patients with COVID-19 presenting to emergency departments: a derivation and validation study. *CMAJ Open* **10**, E90–E99 (2022).
267.
Xiong, F. *et al.* The clinical classification of patients with COVID-19 pneumonia was predicted by Radiomics using chest CT. *Medicine (Baltimore)* **100**, e25307 (2021).
268.
El-Kassas, M. *et al.* The COVEG score to predict severity and mortality among hospitalized patients with COVID-19. *J Infect Dev Ctries* **16**, 1138–1147 (2022).
269.
He, F., Page, J. H., Weinberg, K. R. & Mishra, A. The Development and Validation of Simplified Machine Learning Algorithms to Predict Prognosis of Hospitalized Patients With COVID-19: Multicenter, Retrospective Study. *J Med Internet Res* **24**, e31549 (2022).
270.
Varol, Y. *et al.* The impact of charlson comorbidity index on mortality from SARS-CoV-2 virus infection and A novel COVID-19 mortality index: CoLACD. *Int J Clin Pract* **75**, e13858 (2021).
271.
Ward, D. V. *et al.* The Intestinal and Oral Microbiomes Are Robust Predictors of Covid-19 Severity the Main Predictor of Covid-19-Related Fatality. 2021.01.05.20249061 Preprint at https://doi.org/10.1101/2021.01.05.20249061 (2021).
272.
Kostakis, I. *et al.* The performance of the National Early Warning Score and National Early Warning Score 2 in hospitalised patients infected by the severe acute respiratory syndrome coronavirus 2 (SARS-CoV-2). *Resuscitation* **159**, 150–157 (2021).
273.
Lian, Z. *et al.* The Prediction Model of Risk Factors for COVID-19 Developing into Severe Illness Based on 1046 Patients with COVID-19. *Emerg Med Int* **2021**, 7711056 (2021).
274.
Szabó, I. V. *et al.* The Predictive Role of Artificial Intelligence-Based Chest CT Quantification in Patients with COVID-19 Pneumonia. *Tomography* **7**, 697–710 (2021).
275.
Wang, J. *et al.* Thrombo-inflammatory features predicting mortality in patients with COVID-19: The FAD-85 score. *J Int Med Res* **48**, 300060520955037 (2020).
276.

Dinar, A. M. *et al.* Towards Automated Multiclass Severity Prediction Approach for COVID-19 Infections Based on Combinations of Clinical Data. *Mobile Information Systems* **2022**, e7675925 (2022).
277.
Li, Z. *et al.* Two novel nomograms based on inflammatory cytokines or lymphocyte subsets to differentially diagnose severe or critical and Non-Severe COVID-19. *Aging (Albany NY)* **13**, 17961–17977 (2021).
278.
Ciccarelli, M. *et al.* Untargeted lipidomics reveals specific lipid profiles in COVID-19 patients with different severity from Campania region (Italy). *Journal of Pharmaceutical and Biomedical Analysis* **217**, 114827 (2022).
279.
Bennouar, S. *et al.* Usefulness of biological markers in the early prediction of corona virus disease-2019 severity. *Scandinavian Journal of Clinical and Laboratory Investigation* **80**, 611–618 (2020).
280.
Hoang-Thi, T.-N. *et al.* Usefulness of Hospital Admission Chest X-ray Score for Predicting Mortality and ICU Admission in COVID-19 Patients. *J Clin Med* **11**, 3548 (2022).
281.
Ikemura, K. *et al.* Using Automated Machine Learning to Predict the Mortality of Patients With COVID-19: Prediction Model Development Study. *J Med Internet Res* **23**, e23458 (2021).
282.
Cheng, F.-Y. *et al.* Using Machine Learning to Predict ICU Transfer in Hospitalized COVID-19 Patients. *Journal of Clinical Medicine* **9**, 1668 (2020).
283.
Bradley, P., Frost, F., Tharmaratnam, K. & Wootton, D. G. Utility of established prognostic scores in COVID-19 hospital admissions: multicentre prospective evaluation of CURB-65, NEWS2 and qSOFA. *BMJ Open Respiratory Research* **7**, e000729 (2020).
284.
Assaf, D. *et al.* Utilization of machine-learning models to accurately predict the risk for critical COVID-19. *Intern Emerg Med* **15**, 1435–1443 (2020).
285.
Huang, H.-F. *et al.* Validated tool for early prediction of intensive care unit admission in COVID-19 patients. *World J Clin Cases* **9**, 8388–8403 (2021).
286.
Bello-Chavolla, O. Y. *et al.* Validation and repurposing of the MSL-COVID-19 score for prediction of severe COVID-19 using simple clinical predictors in a triage setting: The Nutri-CoV score. *PLOS ONE* **15**, e0244051 (2020).
287.
Kimura-Sandoval, Y. *et al.* Validation of Chest Computed Tomography Artificial Intelligence to Determine the Requirement for Mechanical Ventilation and Risk of Mortality in Hospitalized Coronavirus Disease-19 Patients in a Tertiary Care Center In Mexico City. *Rev Invest Clin* (2020) doi:10.24875/RIC.20000451.
288.
Shi, Y. *et al.* Validation of pneumonia prognostic scores in a statewide cohort of hospitalised patients with COVID-19. *Int J Clin Pract* **75**, e13926 (2021).

289.
Anurag, A. & Preetam, M. Validation of PSI/PORT, CURB-65 and SCAP scoring system in COVID-19 pneumonia for prediction of disease severity and 14-day mortality. *The Clinical Respiratory Journal* **15**, 467–471 (2021).
290.
Berzuini, C. *et al.* Value of dynamic clinical and biomarker data for mortality risk prediction in COVID-19: a multicentre retrospective cohort study. *BMJ Open* **10**, e041983 (2020).

Supplementary Reference S2. List of review studies evaluated.

1.
Singh, K. *et al.* A meta-analysis of SARS-CoV-2 patients identifies the combinatorial significance of D-dimer, C-reactive protein, lymphocyte, and neutrophil values as a predictor of disease severity. *Int J Lab Hematol* **43**, 324–328 (2021).
2.
Zinellu, A. & Mangoni, A. A. A systematic review and meta-analysis of the association between the neutrophil, lymphocyte, and platelet count, neutrophil-to-lymphocyte ratio, and platelet-to-lymphocyte ratio and COVID-19 progression and mortality. *Expert Rev Clin Immunol* **18**, 1187–1202 (2022).
3.
Toraih, E. A. *et al.* Association of cardiac biomarkers and comorbidities with increased mortality, severity, and cardiac injury in COVID-19 patients: A meta-regression and decision tree analysis. *J Med Virol* **92**, 2473–2488 (2020).
4.
Ji, P. *et al.* Association of elevated inflammatory markers and severe COVID-19: A meta-analysis. *Medicine (Baltimore)* **99**, e23315 (2020).
5.
Malik, P. *et al.* Biomarkers and outcomes of COVID-19 hospitalisations: systematic review and meta-analysis. *BMJ Evid Based Med* **26**, 107–108 (2021).
6.
Katzenschlager, S. *et al.* Can we predict the severe course of COVID-19 - a systematic review and meta-analysis of indicators of clinical outcome? *PLoS One* **16**, e0255154 (2021).
7.
Xie, J. *et al.* Clinical characteristics, laboratory abnormalities and CT findings of COVID-19 patients and risk factors of severe disease: a systematic review and meta-analysis. *Ann Palliat Med* **10**, 1928–1949 (2021).
8.
Meng, Y. *et al.* Clinical Features and Laboratory Examination to Identify Severe Patients with COVID-19: A Systematic Review and Meta-Analysis. *Biomed Res Int* **2021**, 6671291 (2021).
9.
Bao, J. *et al.* Comparative analysis of laboratory indexes of severe and non-severe patients infected with COVID-19. *Clin Chim Acta* **509**, 180–194 (2020).
10.
Wang, L. *et al.* CRP, SAA, LDH, and DD predict poor prognosis of coronavirus disease (COVID-19): a meta-analysis from 7739 patients. *Scand J Clin Lab Invest* **81**, 679–686 (2021).
11.
Soraya, G. V. & Ulhaq, Z. S. Crucial laboratory parameters in COVID-19 diagnosis and prognosis: An updated meta-analysis. *Med Clin (Barc)* **155**, 143–151 (2020).
12.
Elshazli, R. M. *et al.* Diagnostic and prognostic value of hematological and immunological markers in COVID-19 infection: A meta-analysis of 6320 patients. *PLoS One* **15**, e0238160 (2020).
13.
Khodeir, M. M. *et al.* Early prediction keys for COVID-19 cases progression: A meta-analysis. *J Infect Public Health* **14**, 561–569 (2021).
14.
Fang, X. *et al.* Epidemiological, comorbidity factors with severity and prognosis of COVID-19: a systematic review and meta-analysis. *Aging (Albany NY)* **12**, 12493–12503 (2020).
15.

Martins-Filho, P. R., Tavares, C. S. S. & Santos, V. S. Factors associated with mortality in patients with COVID-19. A quantitative evidence synthesis of clinical and laboratory data. *Eur J Intern Med* **76**, 97–99 (2020).
16.
Del Sole, F. *et al.* Features of severe COVID-19: A systematic review and meta-analysis. *Eur J Clin Invest* **50**, e13378 (2020).
17.
Henry, B. M., de Oliveira, M. H. S., Benoit, S., Plebani, M. & Lippi, G. Hematologic, biochemical and immune biomarker abnormalities associated with severe illness and mortality in coronavirus disease 2019 (COVID-19): a meta-analysis. *Clin Chem Lab Med* **58**, 1021–1028 (2020).
18.
Hariyanto, T. I. *et al.* Inflammatory and hematologic markers as predictors of severe outcomes in COVID-19 infection: A systematic review and meta-analysis. *Am J Emerg Med* **41**, 110–119 (2021).
19.
Ghahramani, S. *et al.* Laboratory features of severe vs. non-severe COVID-19 patients in Asian populations: a systematic review and meta-analysis. *Eur J Med Res* **25**, 30 (2020).
20.
Li, J. *et al.* Meta-analysis investigating the relationship between clinical features, outcomes, and severity of severe acute respiratory syndrome coronavirus 2 (SARS-CoV-2) pneumonia. *Am J Infect Control* **49**, 82–89 (2021).
21.
Wungu, C. D. K. *et al.* Meta-analysis of cardiac markers for predictive factors on severity and mortality of COVID-19. *Int J Infect Dis* **105**, 551–559 (2021).
22.
Jain, V. & Yuan, J.-M. Predictive symptoms and comorbidities for severe COVID-19 and intensive care unit admission: a systematic review and meta-analysis. *Int J Public Health* **65**, 533–546 (2020).
23.
Mudatsir, M. *et al.* Predictors of COVID-19 severity: a systematic review and meta-analysis. *F1000Res* **9**, 1107 (2020).
24.
Shi, C. *et al.* Predictors of mortality in patients with coronavirus disease 2019: a systematic review and meta-analysis. *BMC Infect Dis* **21**, 663 (2021).
25.
T, Y., Y, L., Y, Y. & Z, L. Prevalence of comorbidity in Chinese patients with COVID-19: systematic review and meta-analysis of risk factors. *BMC infectious diseases* **21**, (2021).
26.
Sheth, A., Modi, M., Dawson, D. & Dominic, P. Prognostic value of cardiac biomarkers in COVID-19 infection. *Sci Rep* **11**, 4930 (2021).
27.
Wu, Y. *et al.* Risk factors for mortality of coronavirus disease 2019 (COVID-19) patients during the early outbreak of COVID-19: a systematic review and meta-analysis. *Ann Palliat Med* **10**, 5069–5083 (2021).
28.
Zhang, J. J. Y., Lee, K. S., Ang, L. W., Leo, Y. S. & Young, B. E. Risk Factors for Severe Disease and Efficacy of Treatment in Patients Infected With COVID-19: A Systematic Review, Meta-Analysis, and Meta-Regression Analysis. *Clin Infect Dis* **71**, 2199–2206 (2020).
29.
Zheng, Z. *et al.* Risk factors of critical & mortal COVID-19 cases: A systematic literature review and meta-analysis. *J Infect* **81**, e16–e25 (2020).
